# deMULTIplex2: robust sample demultiplexing for scRNA-seq

**DOI:** 10.1186/s13059-024-03177-y

**Published:** 2024-01-30

**Authors:** Qin Zhu, Daniel N. Conrad, Zev J. Gartner

**Affiliations:** 1 Department of Pharmaceutical Chemistry, University of California San Francisco, San Francisco, CA 94158 USA; 2 Chan Zuckerberg Biohub, San Francisco, CA 94158 USA; 3 Center for Cellular Construction, University of California, San Francisco, CA 94158 USA

**Keywords:** scRNA-seq, Sample multiplexing, Demultiplex, Generalized linear models, Expectation–maximization

## Abstract

Sample multiplexing enables pooled analysis during single-cell RNA sequencing workflows, thereby increasing throughput and reducing batch effects. A challenge for all multiplexing techniques is to link sample-specific barcodes with cell-specific barcodes, then demultiplex sample identity post-sequencing. However, existing demultiplexing tools fail under many real-world conditions where barcode cross-contamination is an issue. We therefore developed deMULTIplex2, an algorithm inspired by a mechanistic model of barcode cross-contamination. deMULTIplex2 employs generalized linear models and expectation–maximization to probabilistically determine the sample identity of each cell. Benchmarking reveals superior performance across various experimental conditions, particularly on large or noisy datasets with unbalanced sample compositions.

**Supplementary Information:**

The online version contains supplementary material available at 10.1186/s13059-024-03177-y.

## Background

Single-cell sequencing has revolutionized biomedical research by providing an unbiased, high-resolution, and high-throughput profile of healthy and diseased tissues [1]. Recent advances in single-cell sample multiplexing technologies, such as those based on lipid-tagged indices [2], barcoded antibodies [3–5], chemical labeling [6], nuclear hashing [7], lentiviral infection [8], transient transfection [9], and genetic variation [10–13], further improves the scalability of scRNA-seq, allowing multiple samples from different experimental condition to be pooled together and sequenced. These procedures greatly reduce experimental costs and batch effects while increasing cell throughput, but require demultiplexing of the data to assign each cell to the correct sample-of-origin. In an ideal experiment with samples labeled by lipid- or cholesterol-modified oligos (LMO/CMOs) or antibody-derived tags (ADTs) (both referred to here as “tags”), cells from each sample will be uniquely labeled by only a single tag, and subsequent demultiplexing based on the tag count is trivial. In reality, however, ambient or debris-bound tags may bind to or co-encapsulate with cells from other samples when pooled. These contaminating tags, along with variation in tag capture rate and the inherent technical noise of single-cell sequencing technology, manifest in real data as large numbers of off-target tags (noise) associated with each cell in addition to the on-target tags (signal). The signal-to-noise ratio can vary significantly between cell types and samples, complicating the essential task of identifying a clean cutoff between cells from different samples.

To address this challenge, several computational approaches have been implemented. The deMULTIplex R package, which was released together with MULTI-seq [2], assumes that the positive and negative cells for each tag follow a bimodal distribution, and uses local maxima of a smoothed probability density function (PDF) and quantile sweep to define the threshold for each tag. Similar to the bimodal distribution assumption, GMM-Demux [14] fits a Gaussian mixture model to the tag count data, and uses Bayesian estimation to determine the sample identity of each cell. BFF is another method which was developed based on the bimodal distribution assumption, and offers two modes of classification, one based on raw count (BFF_raw_) and one based on normalized counts (BFF_cluster_). The HashedDrops function in the R package DropletUtils [15] is a straightforward method which assigns each cell to a sample based on its most abundant tag, and uses the log fold change between the highest and second-highest tag counts to represent the confidence of assignment. The HTODemux function in the Seurat package first clusters cells in the tag count space, and then uses the cluster with lowest average tag abundance to fit a negative binomial distribution to define the threshold of calling positive cells [3]. DemuxEM first estimates the background count distribution using empty droplets, then applies the expectation–maximization (EM) algorithm to determine the fraction of a cell’s tag signal coming from the background or the true staining, and performs classification on the background-subtracted data [4]. Lastly, a recent method, demuxmix, uses regression mixture models to account for the positive association between tag count and the number of detected genes, leading to improved classification [16]. These algorithms all rely on some specific feature of the tag count distribution, such as the bimodal distribution, the enrichment of tag in positively labeled cells, or the association between tag count and gene count, to identify a decision boundary in the relevant feature space. However, these assumptions do not fully account for the fundamental physical mechanisms through which distinct tag distributions arise across droplet-based scRNA-seq data. As a consequence, they fail when basic assumptions are not met—such as when sample composition is unbalanced, or tag cross-contamination is high.

Here, we introduce deMULTIplex2, which models tag cross-contamination in a multiplexed single-cell experiment based on the physical mechanism through which tag distributions arise in populations of droplet-encapsulated cells. We first derive the analytical form of the expected tag count, and show that for each tag, the count distribution of cells positively stained by the tag and cells contaminated by the tag are highly distinct in two feature spaces. This allowed us to robustly model these distributions by fitting two negative binomial generalized linear models (GLM-NB) in the corresponding space, and probabilistically determine if a cell is positively labeled by a tag using EM. The distribution of randomized quantile residuals (RQR) suggests that this model fits well on both simulated data and real data with different degrees of noise. When benchmarking deMULTIplex2 against existing methods, we were able to classify significantly more cells with high precision, and the method performs consistently well on noisy, large-scale scRNA-seq datasets generated with diverse multiplexing technologies.

## Results

### Modeling tag cross-contamination

During a single-cell multiplexing experiment, cells are first incubated (labeled) with a sample-specific tag, and then pooled together for single-cell capture and sequencing. The initial labeling of cells in each sample may have variable conditions (i.e., tag concentration, staining time, debris which sequesters tags), but the contamination happens only after pooling when all cells are in the same solution. Therefore, we focus on modeling the contamination process post pooling, which we assume occurs under the same condition across all the cells regardless of which sample they came from because all cells are bathed in the same buffer solution.

**Fig. 1:**
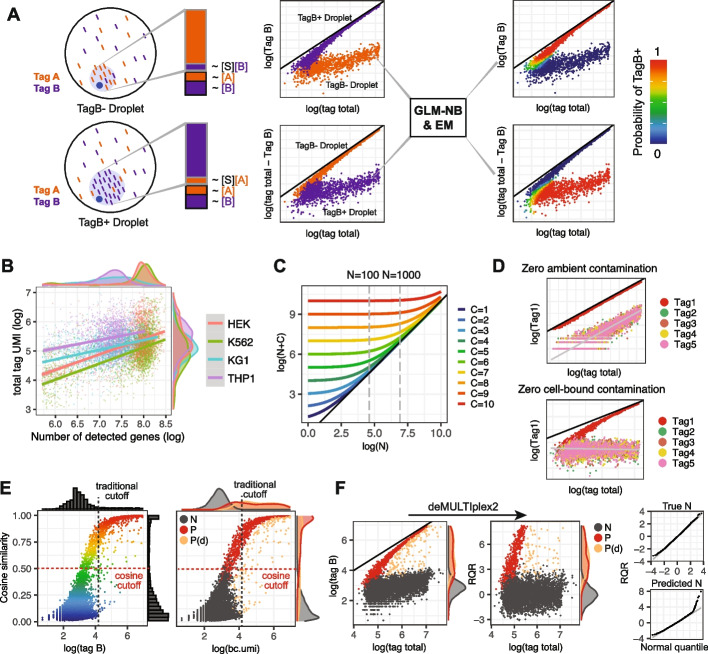
An overview of the deMULTIplex2 algorithm. **A** Illustration for how deMULTIplex2 models tag cross-contamination in a simple two-sample multiplexing experiment. Each sample is labeled with tag A or B. Pooling the samples for single-cell capture allows floating tags to be bound to the cell or captured by the droplet. These contaminating tag counts are modeled by fitting two GLM-NB models in two separate spaces, and the sample identity is inferred using the EM algorithm. The *y* = *x* line is shown in black for this panel and panels **C**, **D**, **F**. **B** Scatter plot illustrating the association between total tag UMI count and number of detected genes in the 4-cell line dataset from Stoeckius et al. [3]. **C** Plot of the relationship between log(N + 𝐶) and log(N) across different values of 𝐶. For a typical experiment, the total tag count of cells is enriched within a range of one to two orders of magnitude (such as in the range of 100 to 1000 highlighted by the dashed line). **D** Simulated tag count distribution for scenarios with zero ambient contamination or zero cell-bound contamination. **E** Cosine similarity with canonical vector plotted against the tag count for a given tag (tag B) using simulated data. Several existing methods look for a bimodal distribution in the count dimension, while deMULTIplex2 takes advantage of the separation between positive and negative cells in the cosine dimension to initialize EM. **F** (left) The original UMI count of a given tag plotted against total tag count with simulated data. **F** (right) RQRs computed by deMULTIplex2 of the same simulated data plotted against total tag counts. N: negative cells, P: positive cells, P(d): doublets positive with tag B. The RQRs are plotted against the normal quantiles for true negative cells and predicted negative cells

Consider a simple experiment consisting of two samples, labeled with tags A and B respectively (Fig. 1A). After the samples are pooled together, excess tag B can bind to cells that were initially labeled with tag A (tagA + /tagB − cells) and vice versa, causing contamination. We model this process as a simple chemical reaction between the cell surface S and tag B:

$$S+B \stackrel{ k1 }{\to } SB,$$

where $k1$ is the rate constant of the reaction. Typically, the reaction will not reach equilibrium because there will be limited incubation time at low temperature prior to single-cell capture (as recommended by most protocols). Therefore, the “concentration” of cell-bound tag B, as denoted by [SB], can be expressed as

1 $$\left[SB\right]=k1\left[S\right]\left[B\right]t={K}_{B}\left[S\right].$$

${K}_{B}$ is assumed to be a constant which is uniform across all the cells because of the same binding mechanism ($k1$), ambient concentration ($\left[B\right]$), and incubation time ($t$).

This suggests that the final bound tag count is proportional to the total cell surface area, and larger cells tend to get more bound tags. Indeed, when plotting the total number of bound tags against the total number of genes (which is typically considered to be correlated with cell size), positive correlations are observed across different cell types (Fig. 1B) [3]. The demuxmix method builds upon this observation to fit a regression mixture model with total detected gene count as a predictor to account for the extra variance observed in tag count data [16]. However, gene count is highly cell-type-specific and can lead to cell-type-biased classifications (Fig. 1B). On the other hand, the total tag count $${N}_{total}$$ exhibits much less cell-type specificity, and therefore may be more associated with cell surface area (Fig. 1B), i.e. $${N}_{total}\propto [S]$$. This additional assumption allows us to re-write Eq. 1 to represent the expected tag count of contaminating B ($${\mu }_{B})$$ as a fraction ($${p}_{B})$$ of total tag counts:

2 $${\mu }_{B}={p}_{B}{N}_{total}.$$

Due to sampling variation inherent to scRNA-seq technology, the observed unique molecular identifier (UMI) count is commonly modeled with a Poisson or Negative binomial (NB) distribution [17]. In practice, we and others have observed overdispersion in the tag UMI count data [3, 16]. We therefore choose to use a negative binomial distribution and fit the observed tag counts ${X}_{B}$ with a generalized linear model:

3 $${X}_{B} \sim NB({\mu }_{B},{\theta }_{B})$$

4 $${{\text{ln}}(\mu }_{B})={{\text{ln}}(p}_{B})+{\text{ln}}\left({N}_{total}\right).$$

Equations 2– 4 are similar to a proposed analytical solution of scRNA-seq UMI count distributions [18]. As Lause et al. pointed out [18], this model suggests that the linear coefficient ${\beta }_{1}$ before ${\text{ln}}({N}_{total})$ should be fixed to 1 for negative control cells without biological variability (in this case, cells that have not been labeled with the contaminating tag prior to pooling). However, although the fit of ${\beta }_{1}$ for some real datasets is indeed very close to 1, the majority of datasets we have tested demonstrate an estimated ${\beta }_{1}$ lower than 1 (Additional file 1: Fig. S1A). When inspecting the count distributions of these datasets; however, we realized that there is a second source of contamination, where ambient floating tags or debris-bound tags are co-encapsulated in the droplet and get sequenced along with the cell-surface-bound tags (Fig. 1A, Additional file 1: Fig. S1). Under such a model, the expected UMI count of ambient B (denote as ${M}_{B}$) is constant across cells assuming consistent droplet size and is proportional to the concentration of ambient B. Combining these two sources of contaminating tags, Eq. 2 for the expected count of contaminating tag B should be revised as:

5 $${\mu }_{B}={p}_{B}\left({N}_{total}- \sum_{k=1}^{n}{M}_{k}\right)+ {M}_{B}={p}_{B}{(N}_{total}+C).$$

Note that ${p}_{B}\left({N}_{total}- \sum_{k=1}^{n}{M}_{k}\right)$ is representing the cell-bound contamination previously defined by Eq. 2, but with the total cell-bound tag count re-calculated by excluding the sum of all ambient tag count (${M}_{k}$ over $n$ tags) from the total observed tag count. $C=\frac{{M}_{B}}{{p}_{B}}-\sum_{k=1}^{n}{M}_{k},$ which is the same across all negative cells contaminated with tag B and does not depend on the total observed tag count, thus can be treated as a constant. Therefore,

6 $${\text{ln}}\left({\mu }_{B}\right)={\text{ln}}\left({N}_{total}+C\right)+{\text{ln}}\left({p}_{B}\right).$$

In the above equation, the relationship between ${\text{ln}}\left({\mu }_{B}\right)$ and ${\text{ln}}\left({N}_{total}\right)$ is no longer linear. $C$ is a tag-specific constant which is difficult to estimate. However, looking at the relationship between ${\text{ln}}\left({N}_{total}+C\right)$ and ${\text{ln}}\left({N}_{total}\right)$ across different values of $C$, we found within a limited range of ${\text{ln}}\left({N}_{total}\right)$, such as that typically observed in total tag counts (one or two orders of magnitude of difference in total tag count, likely limited by the size range of eukaryotic cells, Fig. 1B), their relationship is approximately linear (Fig. 1C), i.e.,

7 $${\text{ln}}\left({N}_{total}+C\right)\approx {\beta }_{1}{\text{ln}}\left({N}_{total}\right)+{\beta }_{0}.$$

Then

8 $${\text{ln}}\left({\mu }_{B}\right)={\beta }_{1}^{neg}{\text{ln}}\left({N}_{total}\right)+{\beta }_{0}^{neg}.$$

Here, ${\beta }_{1}^{neg}$ and ${\beta }_{0}^{neg}$ are linear coefficients that can be estimated with a GLM-NB model. Modeling these two sources of contamination allows us to simulate datasets with different ratios of cell-bound and ambient tag contamination. Encouragingly, simulated data qualitatively reproduces a variety of distributions we see in real datasets (Fig. 1D, Additional file 1: Fig. S1).

To specify the full probabilistic model for the data, we also need to model the count distributions of the positive cells which were originally labeled with tag B. As discussed before, we cannot directly model the count of tag B prior to pooling. But in the simple experiment illustrated in Fig. 1A, positive cells of B are also the negative cells of A, meaning that the same GLM-NB model described by Eqs. 3 and 8 can be applied to the tag count of A (${{X}_{A}=N}_{total}-{X}_{B}$) to model the distribution of the positive cells of B.

More generally, for the positive cells labeled with a particular tag B, we consider the distribution of total contamination count ${N}_{total}-{X}_{B}$, where

9 $${N}_{total}-{X}_{B}= \sum_{k\ne B}{X}_{k}.$$

Assuming the counts of each of the contaminating tags follow a NB distribution, the total contamination is the convolution of multiple NB distributions, which also has the form of a NB distribution having a mean equal to the sum of all means of contaminating tags:

10 $$\sum_{k\ne B}{X}_{k}\sim NB\left(\sum_{k\ne B}{\mu }_{k}, {\theta }^{pos}\right).$$

This result makes intuitive sense because the tags share the same chemical and physical properties, so the pool of contaminating tags can be thought as a single contaminating meta-tag. Following derivation similar to that for single-tag contamination, the expected tag count for multi-tag contamination follows:

11 $${\text{ln}}\left(\sum_{k\ne B}{\mu }_{k}\right)={\beta }_{1}^{pos}{\text{ln}}\left({N}_{total}\right)+{\beta }_{0}^{pos}.$$

We provide the detailed derivation of (11) in “ Methods.” Eqs. 9– 11 suggest that the distribution of positive cells can be modeled with a second GLM-NB model in the ${N}_{total}-X$ vs ${N}_{total}$ (total tag count minus observed count of positive tag vs. total tag count) space (Fig. 1A). It is important to point out that the distribution of positive cells in the $X$ vs ${N}_{total}$ space (positive tag count vs total tag count) is non-linear with ambient contamination. The cells converge to the *y* = *x* line with increased signal-to-noise ratio, but can never cross the *y* = *x* line (Fig. 1A, D). Therefore, regression mixture models, such as that proposed by demuxmix [16], cannot properly fit positive cells in this space and will likely result in poor classification when ambient contamination is present.

### Probabilistic classification of cells with expectation–maximization

The two GLM-NB models specified in the separate spaces allow us to define the joint probability distribution of all cells and use expectation–maximization (EM) to solve for the identity (positive or negative) of each cell for each tag. The joint probability distribution for each tag can be expressed as:

12 $$p\left(X,Z \right|\Theta )= \prod_{i=1}^{N}p({X}_{i}, {Z}_{i})= \prod_{i=1}^{N-r}p\left({X}_{i}|{Z}_{i}=0\right)\prod_{j=1}^{r}p\left({X}_{j}|{Z}_{j}=1\right).$$

Here, $N$ is the total number of cells, $r$ is the number of positive cells, and the latent variable ${Z}_{i}$ indicates whether a cell $i$ is positively labeled by the tag. For each tag *T* and each cell *i*, the conditional probability follows the NB distribution derived in the previous section, i.e.,

13 $${X}_{i}\sim NB\left({\mu }_{T}, {\theta }^{neg}\right), if \,{Z}_{i}=0$$

14 $${N}_{total}-{X}_{j}\sim NB\left(\sum_{k\ne T}{\mu }_{k}, {\theta }^{pos}\right), if \,{Z}_{j}=1$$

The EM algorithm iterates between estimating each cell’s identity, as described by $Z$, and fitting the two GLM-NB model in the corresponding spaces. The algorithm stops upon convergence, or when the user-specified maximum number of iterations has been reached. In practice, we found that for most datasets the algorithm quickly converges when using a reasonable number of cells for model fitting (Additional file 1: Fig. S2). Because random sampling of a few thousand cells is enough for robust fitting of the GLM-NB models (Additional file 1: Fig. S2), deMULTIplex2 can process any arbitrarily large datasets with high speed, low memory requirement, and robust performance.

Finally, upon convergence, deMULTIplex2 reports the posterior probability of a cell being positively labeled by each tag (“Methods,” Fig. 1A). The decision boundary produced by the algorithm usually has a large margin, with relatively small difference in assignment results from different choice of probability cutoff (Fig. 1A, Additional file 1: Fig. S1). Therefore, deMULTIplex2 can resolve each cell’s identity with high confidence in a probabilistic manner. With each cell being classified as positive or negative for each tag, deMULTIplex2 determines whether a cell is a singlet, a multiplet (generally referred to as “doublet”) or negative (not labeled with any tag) based on the total count of positive tags.

### Initializing EM with cosine similarity cutoff

The EM algorithm is known to be sensitive to initialization [19, 20]. Previous efforts have addressed this issue through multiple randomly initialized short runs [21–23] or through an initial clustering [16, 24]. However, these strategies require additional computation and may still fail due to imbalanced cell number between positive cells and negative cells (which is typical for a multiplexed dataset). Therefore, we sought a statistic, derived from the unique features of positive cells and negative cells, that robustly generates a satisfactory initial separation among positive and negative cells and can properly initialize the EM algorithm.

We found that the cosine similarity between the tag count vector of each cell and the canonical vectors for each tag (i.e., a vector with 1 s and 0 s, where position of 1 indicate which tag the vector represents) provides a close-to-truth initial guess for the identity of each cell. The cosine metric can be understood using a barnyard plot. Assuming low contamination in a two-tag mixture experiment, true positive singlets will be aligned with each axis; the resulting cosine similarity with the canonical vectors < 1,0 > and < 0,1 > will be 1 and 0 or vice versa. In real datasets where true positive tags take up the majority of the tag reads in a cell, the cosine similarity, when plotted against the tag count, approximately follows a sigmoid curve (Fig. 1E). The distribution in the tag count dimension is traditionally used to define a cutoff for positive vs negative cells, and its bimodality is the core assumption of many existing methods. However, in experiments with many pooled samples, imbalanced numbers of cells per sample, or high background noise, the positive peak could be undetectable or could overlap significantly with the negative peak (Fig. 1E), leading to failures in methods that rely heavily on the assumption of bimodality. The cosine similarity, in contrast, provides a second dimension with larger margins for drawing the initial cutoff to initialize the EM (Fig. 1E). It correctly enriches for true positive cells on one side of the sigmoid, and for true negative cells on the other side. With both real data and simulated data, we found that EM initialized with any cosine cutoff in the range of 0.2 to 0.9 will almost always quickly and robustly converges to the correct fit (Additional file 1: Fig. S2).

### Randomized quantile residuals for diagnosing the goodness-of-fit

Residual plots show the discrepancy between data and model and are commonly used to diagnose goodness-of-fit. In a normal linear model, the Pearson residual is defined as ${r}_{i}= \frac{{y}_{i}-\widehat{{\mu }_{i}}}{{V(\widehat{{\mu }_{i}})}^\frac{1}{2}}$, where $\widehat{{\mu }_{i}}$ is the fitted value for random variable ${y}_{i}$ and $V(\widehat{{\mu }_{i}})$ is the estimated variance. The Pearson residual is normally distributed under the true model. However, for generalized linear models for Poisson or NB distributions, the residual is far from normal due to the discrete response values. Randomized quantile residuals (RQRs) were proposed [25] to overcome this problem and have been applied for diagnosing GLM models for count data [26, 27]. RQR is an extension of the quantile residual (QR), which inverts the fitted distribution function of each observation to the corresponding normal quantile. QR is defined as:

15 $${r}_{q, i}= {\Phi }^{-1}\{F\left({y}_{i};\widehat{{\mu }_{i}},\widehat{\phi }\right)\}$$

$F\left({y}_{i};\widehat{{\mu }_{i}},\widehat{\phi }\right)$ is the cumulative distribution function for random variable ${y}_{i}$ with expected value ${\mu }_{i}$ and parameter $\phi$. ${\Phi }^{-1}$ is the quantile function of a standard normal distribution. To generalize QR to the discrete cumulative distribution F of Poisson and NB, a random uniform sampling was performed for each observation to obtain a continuous mapping to the normal distribution, i.e.,

16 $${r}_{q, i}= {\Phi }^{-1}\left({u}_{i}\right)$$

where ${u}_{i}$ is drawn randomly from a uniform distribution defined on the interval $({sup}_{y<{y}_{i}}F({y};\widehat{{\mu }_{i}},\widehat{\phi }), F({y}_{i};\widehat{{\mu }_{i}},\widehat{\phi })]$.

Therefore, under the null hypothesis, the RQR of a well-specified model will be normally distributed.

Using RQR, we evaluated the goodness-of-fit of the two GLM-NB models for the positive cells and negative cells using both simulated data and real data. We plotted the RQRs against the standard normal quantiles, also known as the Q-Q plot, for each regression fit. We found that for true negative cells, RQRs are indeed normally distributed, and for deMULTIplex2-predicted negative cells, the RQRs are very close to normal, and are often right-skewed due to the ambiguity at the boundary of positive and negative cells (Fig. 1F, S1). For positive cells in the ${N}_{total}-X$ vs ${N}_{total}$ space, however, the RQRs deviate from normal for several tags in the real dataset (Additional file 1: Fig. S1), likely due to the presence of doublets. Interestingly, we found the final classification is still very close to the ground truth despite this imperfect fit, likely because the distinct distributions of positive and negative cells in the two spaces lead to large difference between predicted positive probability and negative probability from the two GLM models, and any misclassification during the EM will incur a high cost and will be corrected in the next few EM iterations.

### deMULTIplex2 outperforms other methods on simulated data

**Fig. 2:**
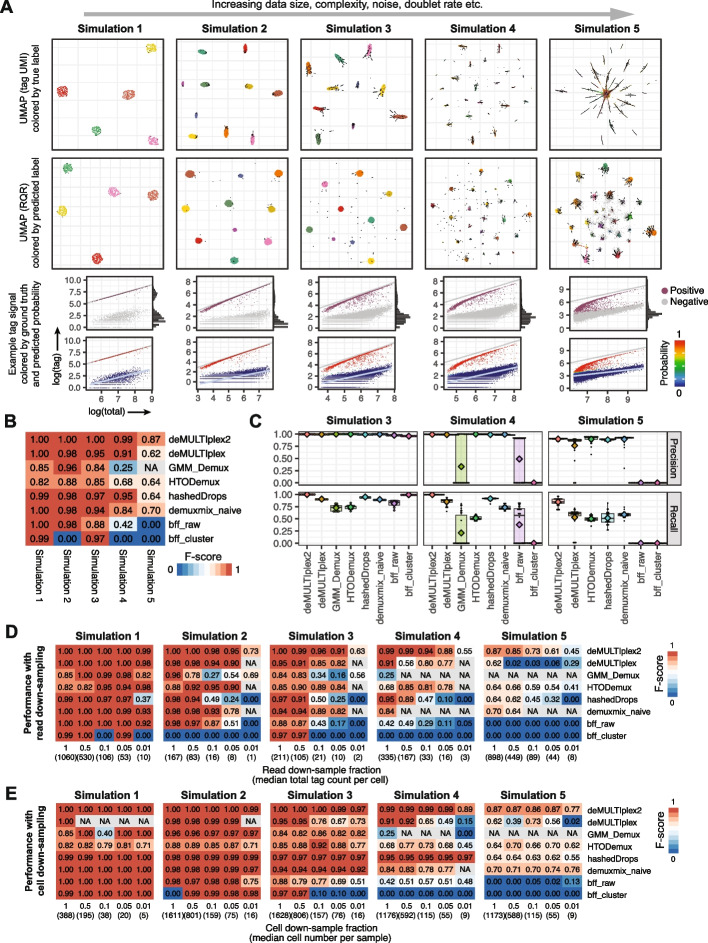
Performance of deMULTIplex2 on simulated datasets. **A** Five simulations with different data size, complexity and noise, as listed in Additional file 2: Table S1. The top row shows UMAPs computed with raw tag UMI count. The middle row shows UMAPs based on deMULTIplex2-computed RQRs. The bottom row shows the cell distribution in the log(tag umi) vs log(total tag count) space of a randomly selected tag in each simulation, colored by ground-truth identity and deMULTIplex2 computed posterior probability of being positive for that tag. The *y* = *x* line (grey) and the GLM-NB fit for the negative cells (steel blue line) are also plotted. **B** Heatmap summarizing the F-score of deMULTIplex2 and other methods on the five simulated datasets. Methods that require mRNA count matrix as input were excluded from this comparison. NA indicates the method failed to run. **C** Per-tag performance of deMULTIplex2 compared to other methods. Mean values are highlighted with the diamond points. **D** Performance of all methods on the simulated datasets with down-sampled reads. Median total tag count per cell is listed for each down-sampling rate. **E** Performance of all methods on the simulated datasets with down-sampled cells. Median cell number per sample is listed for each down-sampling rate

The two-component contamination model allows us to simulate datasets which recapitulate a spectrum of realistic tag distributions (Additional file 2: Table S1, Fig. 2A, Additional file 1: Fig. S1). Among all possible simulations, we selected five conditions with varying data size, complexity and noise covering a wide span of parameter values encountered in real data sets in order to benchmark the performance of demultiplexing algorithms (Additional file 2: Table S1, Fig. 2A,C). Consequently, the resulting UMAPs based on the raw tag UMI count resemble what we often see from real datasets, which have distinct clusters for clean-labeled samples and star-shaped structures for noisy samples. For the latter case, the ambiguous, low-tag-count cells are often located in the center of the star and high-tag-count cells are located at the periphery. Notably, when plotting the UMAP based on deMULTIplex2-computed RQRs, the embedding is much less influenced by the total tag count, and the doublets are placed at the periphery of each cluster or as separate clusters, suggesting that RQR provides a proper normalization of the tag count data in addition to its diagnostic capability.

We used F-score (harmonic mean of precision and recall) as a balanced statistic to represent overall performance. Almost all methods perform relatively well on small and clean dataset (Fig. 2B, Simulation 1). Changes in data composition (sample number, cell number per sample etc.) have limited impact on the performance of most methods when the cross-contamination level is low (Fig. 2B, Simulation 2 vs Simulation 1). Similarly, on small-to-medium-sized data, elevated level of cross-contamination and doublet rate only modestly decreases the accuracy of most methods (Fig. 2B, Simulation 3 vs Simulation 2). Notably, deMULTIplex2 was the only method that maintained its top performance across these conditions (Fig. 2B).

We observed different results in more complex simulations. For example, when the sample number increased from 10 to 30 and imbalance in cell number across samples was increased, a dramatic drop in performance was observed for multiple methods (Simulation 4 vs Simulation 3, Fig. 2B). Methods such as GMM-Demux and BFF completely fail to recover many samples, likely because these methods heavily rely on the assumption of bimodal distribution, which is not a realistic assumption for large datasets (Fig. 2A, bottom panel). Methods such as HTODemux and demuxmix (naïve mode) have a significant drop in recall despite maintaining their precision (Fig. 2C), mainly because these methods were classifying the high-tag-count, high-confidence cells. In theory, achieving high recall without sacrificing precision is much more challenging because higher recall requires the method to draw a decision boundary closer to the ambiguous and negative territory (without crossing the border to incur misclassification). In practice, high recall is often desired because it means many more real singlets can be recovered from expensive single-cell experiments. In this case, deMULTIplex2 was able to correctly identify the close-to-optimal decision boundary (Fig. 2A, bottom panel) and classified > 99% real singlets, while HTOdemux and demuxmix (naïve mode) were only able to achieve an average recall of 52 and 73%, respectively. Upon further increasing the data complexity and noise of the simulation, all methods suffer in performance, but deMULTIplex2 maintained its top performance and has consistent classification accuracy across all samples, even for those with very few cells (Simulation 5, Fig. 2A–C).

For each of these simulated datasets, we performed read down-sampling to benchmark the methods’ robustness to sequencing depth. As shown in Fig. 2D, deMULTIplex2 was consistently the top performer across a range of sequencing depths, with several methods failing when the total tag count per cell became too low and the drop-out rate became too high (Fig. 2D). This suggests that the statistical model powering deMULTIplex2 is robust against low sequencing depth and high drop-out rate. The sequencing depth has most significant impact on performance for large and noisy datasets, suggesting increasing sequencing depth (before saturation) may be beneficial for these types of data.

Finally, we down-sampled the five simulated datasets to retain different number of cells and benchmarked deMULTIplex2’s performance on the down-sampled datasets. Figure 2E shows deMULTIplex2 has maintained its classification accuracy even with 1–5% of original cells (or ~ 10 cells per group), while several other methods exhibit significant deteriorating performance with decreased number of cells. This suggests deMULTIplex2’s model fitting does not heavily rely on the cell number.

### deMULTIplex2 outperforms other methods on real-world datasets

**Fig. 3:**
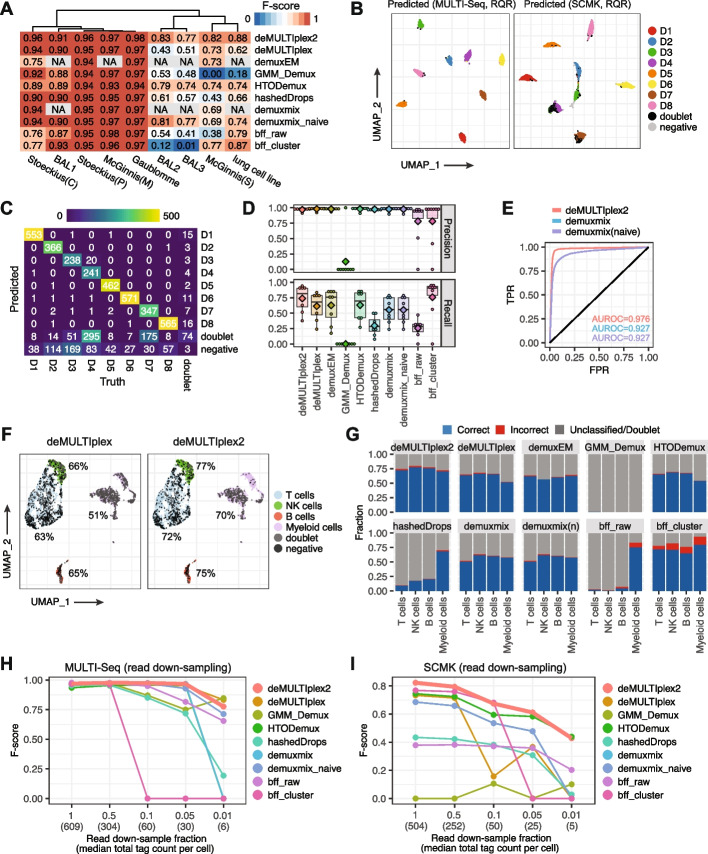
Performance of deMULTIplex2 on real datasets. **A** Heatmap summarizing the F-score of deMULTIplex2 and other methods on 9 real datasets. Stoeckius(C) and Stoeckius(P) are cell line and multi-donor PBMC datasets from Stoeckius et al. [3]. McGinnis(M) and McGinnis(S) are MULTI-seq and SCMK datasets from McGinnis et al. [28]. BAL1, 2, 3 are three batches of multi-donor bronchoalveolar lavage (BAL) datasets from Howitt et al. and Maksimovic et al. [29, 30]. The lung cell line dataset is also from Howitt et al. [30]. NA indicates the method cannot be run on the corresponding datasets due to the unavailability of mRNA count matrix or an error (i.e., demuxEM returns an error on the MULTI-seq PBMC dataset). **B** UMAP computed with deMULTIplex2-computed RQR for the MULTI-seq and SCMK datasets from McGinnis et al. [28], colored by donor ID predicted by deMULTIplex2. **C** Concordance between deMULTIplex2-predicted donor ID and the true donor ID based on SNP-based sample classification using souporcell [12]. **D** Performance of deMULTIplex2 and other methods on each sample. Mean values are highlighted with the diamond points. **E** Multiclass ROC curve of deMULTIplex2 and the two modes of demuxmix. False positive rate (FPR) and true positive rate (TPR) were computed for all samples using a one-vs-rest scheme and averaged to generate the ROC curve. **F** deMULTIplex and deMULTIplex2 recovered cells in the gene expression space. Percentage of correctly classified singlets are highlighted for each of the cell type. **G** Classification accuracy of each cell type across methods. **H** Performance of all methods on the MULTI-Seq dataset from McGinnis et al. [28] with down-sampled reads. demuxEM returns an error and is excluded from this analysis. **I** Performance of all methods on the SCMK dataset from McGinnis et al. [28] with down-sampled reads

We assembled ten real-world datasets with associated ground-truth information to benchmark the performance of deMULTIplex2 and other methods (Additional file 2: Table S2). These datasets include the 8-donor PBMC MULTI-seq and SCMK dataset from McGinnis et al. [28], the 4-cell line and 8-donor PBMC datasets from Stoeckius et al. [3], the 8-donor single-nucleus human brain cortex datasets from Gaublomme et al. [4], the three batches of multi-donor bronchoalveolar lavage (BAL) datasets from Howitt et al. and Maksimovic et al. [29, 30], and the human lung cell line dataset from Howitt et al. [30]. On datasets collected from different donors, SNP-based classification was used to obtain the ground-truth labels [11, 12]. For datasets comprising different cell lines, ground-truth labels were obtained by clustering in the transcriptomic space. As shown in Fig. 3A and Additional file 2: Table S3, deMULTIplex2 consistently demonstrated superior performance in singlet classification, while its performance on doublet calling is comparable to existing methods (Additional file 1: Fig. S3).

Among these datasets, the MULTI-seq dataset from McGinnis et al. [28], and the ADT datasets from Stoeckius et al. [3] and Gaublomme et al. [4] are clean datasets with a very low degree of contamination. Therefore, all methods were able to achieve high precision with only a few exceptions. Notably, deMULTIplex2 was able to achieve highest average recall for most of these datasets, suggesting it can robustly retrieve real singlets without sacrificing precision (Additional file 2: Table S3).

The BAL dataset consists of three separate batches with batch 2 and 3 having higher levels of contamination and doublet rates compared to batch 1 [29, 30]. On the noisy batches, deMULTIplex2 was able to achieve F-scores of about 0.8 with a significant lead over other methods (Additional file 2: Table S3). Similarly, on the lung cell line MULTI-seq dataset and PBMC SCMK dataset, deMULTIplex2 was able to correctly retrieve many more singlets compared to other methods.

Of these datasets, the 8-donor SCMK PBMC dataset was of particular interest to us because the authors labeled the cells with both ADTs from single-cell multiplexing kit (SCMK) reagents (BD Biosciences), and the MULTI-seq LMOs [28]. The authors observed classification of the MULTI-seq tags was much better than classification of the SCMK tags, with the latter showing cell-type biases. We asked if deMULTIplex2 can classify more genuine singlets despite this technological and biological bias. When applying deMULTIplex2 to this dataset, we were able to reproduce the authors’ observation that classification on the MULTI-seq tag count resulted in much better results compared to the SCMK results (Fig. 3B). Compared to previous deMULTIplex-based classification and results from other classification methods, deMULTIplex2 was able to achieve higher recall on the noisy SCMK dataset with high precision (Fig. 3C,D). BFF_cluster_ was able to achieve comparable precision and recall on six donors, but the method performs very poorly on the other two donors with noisy tag data (Fig. 3D). Among these methods, demuxmix also generates probabilistic assignment like deMULTIplex2, allowing us to compare the Receiver Operating Characteristic (ROC) between the two methods. As shown in Fig. 3E, deMULTIplex2 has a much higher area under the ROC curve (AUROC) compared to the two modes of demuxmix, suggesting our mechanism-guided model better captures the difference between positive and negative cell distributions. Looking at the transcriptomic space, we found cell-type bias is still present with deMULTIplex2 classification, but more cells were recovered compared to the deMULTIplex result (Fig. 3F,G). However, when comparing to other results from existing tools, deMULTIplex2 has much lower cell-type bias, and significantly higher classification accuracy (Fig. 3G).

We performed read down-sampling on both the MULTI-seq tag count matrix and the SCMK tag count matrix to examine the effect of sequencing depth on demultiplexing accuracy. For the clean MULTI-seq data, deMULTIplex2 yields close-to-one F-score even with just 5% of the original reads (median 30 tags per cell), while the performance of several other methods drops significantly with decreased tag count (Fig. 3H). On the noisy SCMK dataset, deMULTIplex2 had decreased accuracy at lower sequencing depth, but still outperforms other methods. These observations suggest classification accuracy on noisy data is more dependent on sequencing depth, but for an experiment with low tag cross-contamination, a user can achieve similar classification accuracy with deMULTIplex2 even with shallow sequencing on the tag library.

### deMULTIplex2 can salvage cells from complex experiments using precious samples

Many single-cell experiments are carried out on precious samples with limited source material, such as tumor cells from patients [31] or rare cell populations during development [32]. Using single-cell multiplexing technology on these samples can reduce batch effects, but depending on the sample quality and cell number, the final cell count recovered from each sample may exhibit large variability. Therefore, demultiplexing methods should be able to robustly handle experimental design where the total cell number per sample is variable and maximally salvage cells from low-cell-count samples.

**Fig. 4:**
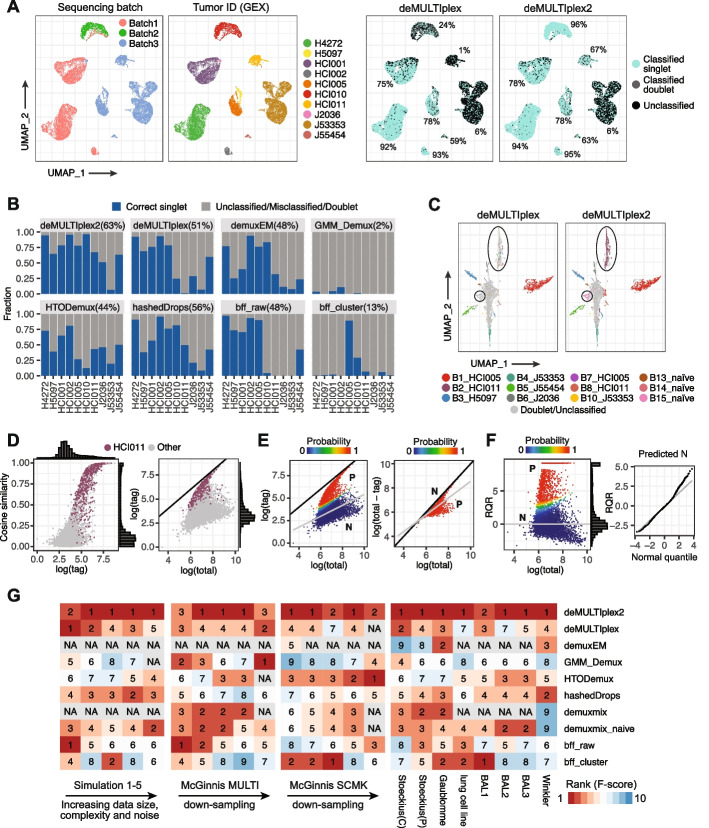
Performance of deMULTIplex2 on multiplexed PDX of human breast cancer. **A** UMAP computed with gene expression (GEX) colored by sequencing batch, expression-based tumor ID, and classification results by deMULTIplex and deMULTIplex2. For major tumor clusters, we highlight the percentage of correctly classified singlets. **B** Fraction of cells correctly predicted by deMULTIplex2 and other methods for each tumor model. All methods were run with default parameters. Demuxmix was excluded from the comparison because it returned errors on all three batches. **C** UMAP of cells from batch 3 computed using raw tag UMI counts. Circles highlight two samples that were missed by deMULTIplex with default settings, but were recovered with deMULTIplex2. **D** Cosine similarity vs. tag count plot and the tag count vs. total tag count plot for the sample tagged with “Bar2” and from tumor “HCI011” recovered by deMULTIplex2. The *y* = *x* line is shown in black. **E** GLM-NB fit (grey line) and posterior probability of cells being positively tagged by tag “Bar2” calculated by deMULTIplex2 in the two modeling spaces. The *y* = *x* line is shown in black, and majority of negative cells fall on or near that line in the second space. N: negative cells, P: positive cells. **F** RQR plotted against log total tag count, colored by posterior probability. For some positive cells, its RQR is infinity. These values were capped to the maximum value of non-infinity RQRs plus 1 for visualization purposes. The Q-Q plot compares the distribution of RQRs of predicted negative cells to that of a normal distribution. **G** Heatmap summarizing the rank of F-score of deMULTIplex2 and other methods on both simulated and real-world datasets. Dataset abbreviations are same as those in Fig. 3A. Winkler is the multiplexed PDX dataset from Winkler et al. [33]. NA indicates the method cannot be run on the corresponding datasets due to the unavailability of mRNA count matrix or an error

To understand tumor metastasis in breast cancer, Winkler et al. performed MULTI-seq on a large panel of patient-derived xenograft models (PDX) of human breast cancer [33]. The collection and sequencing of the tumors were done across three batches, with the tumors being too heterogeneous to be easily separated in gene expression space (Fig. 4A). The three batches comprise multiple samples of varying total cell numbers, with some samples having very few tagged cells. When applying existing demultiplexing methods on this dataset, several methods, including demuxmix, GMM-Demux, and HTODemux, report errors on one or all of the batches, likely due to the samples with low cell number. Although the rest of the methods were able to classify all three batches without reporting errors, their performance was inferior to deMULTIplex2 (Fig. 4B). In the end, deMULTIplex2 was able to correctly retrieve the highest number of real singlets (63.2% of all cells) compared to deMULTIplex (50.8% of all cells), demonstrating an approximate 25% performance increase. Notably, deMULTIplex2 was able to recover two PDX samples that were almost completely missed by deMULTIplex, GMM-Demux, and BFF (Fig. 4C). Examining the tag count distribution of one of such sample (HCI011 tumor tagged with tag “Bar2”), we did not observe a clear bimodal distribution, which likely contributes to failure for methods that rely on such an assumption (Fig. 4D). However, in the axis of cosine similarity, the true positive cells and negative cells are well separated with a much more apparent bimodal distribution (Fig. 4D). With the two GLM-NBs fitted in the two separate spaces, deMULTIplex2 was able to correctly recover 70% of HCI011 tumor cells from the sample (the batch contains another HCI011 sample with a different tag, so the actual recall may be even higher) (Fig. 4E). Finally, when checking the RQRs, we found the distribution of predicted negative cells is close to normal but has a heavy right tail, similar to what we observed with noisy, simulated data (Fig. 4F, Additional file 1: Fig. S1C). Majority of the predicted positive cells deviate significantly from the negative cell distribution in the residual plot, and there are few cells with ambiguous posterior probability near 0.5 (Fig. 4F).

Thus, deMULTIplex2 can properly handle complex multiplexed scRNA-seq experiments with precious samples, recover noisy sample tags from these experiments, and does not require the users to pre-filter the tags based on cell number and sample quality. By doing so, it significantly improves the quality of downstream analyses that depend on cell number, such as differential gene expression analysis.

Summarizing results from all benchmarking analyses, we found that deMULTIplex2 is consistently top-performing across different biological samples, technologies, sequencing depths, and contamination levels (Fig. 4G). Therefore, without prior knowledge regarding these experimental parameters, deMULTIplex2 should be the method of choice for robust and accurate sample demultiplexing.

### Improvements on speed and memory

The deMULTIplex package was designed as a complete demultiplexing pipeline which starts from the preprocessing of raw tag FASTQ files [2]. In deMULTIplex2, we have overhauled the code to improve preprocessing steps. Specifically, deMULTIplex2 utilizes the sparse matrix data structure to efficiently tabulate the tag count of each cell, greatly accelerating computation and reducing the required memory.

As shown previously, the classification algorithm of deMULTIplex2 is highly robust even with down-sampling of cells. Therefore, when running on large single-cell datasets, deMULTIplex2 performs down-sampling by default when fitting the GLM-NBs. A 100,000 single-cell dataset can be classified by deMULTIplex2 on a MacBook in only a couple of minutes. The software also outputs publication-quality summary and diagnostic plots for users to examine the results in detail.

## Discussion

Although many existing demultiplexing methods work well on small and clean multiplexed datasets, their performance deteriorates rapidly when processing data with large numbers of samples and with noise arising from cross-contamination of tags. Such datasets have become more common with the increasing throughput of single-cell platforms, which can best be leveraged using multiplexing technologies. This motivated us to develop deMULTIplex2, which is built on a statistical model of tag count distributions derived from the physical mechanism of the contamination process. We found that by modeling contamination from two sources, the cell-bound contamination and the ambient contamination, the observed tag distribution from many real-world experiments can be recapitulated. Using generalized linear models and EM, we were able to probabilistically infer the sample identity of each cell and classify each cell with high confidence. Using real and simulated datasets, we demonstrated that deMULTIplex2 significantly outperforms other methods in recovering genuinely tagged singlets without compromising precision. This improvement in performance is particularly valuable for real-world applications, as many multiplexed scRNA-seq experiments are carried out on precious samples with limited number of starting cells. More broadly, real-world datasets often suffer from higher background and barcode variability that hinders sample classification using previously reported algorithms. Methods that were able to achieve high precision often fail to recover the majority of the true singlets, because they are only classifying cells with high signal-to-noise ratio. Similarly, methods that have high cell recovery often have low precision, as they are misclassifying cells with noisy tag signals. deMULTIplex2 enables recovery of significantly more cells from these samples without sacrificing classification accuracy, as it is able to correctly draw the decision boundary to separate true positive cells and negative cells. Encouragingly, this behavior is seen consistently across simulated datasets with varying degree of noise and complexity, as well as real-world datasets generated with multiple multiplexing technologies and comprising diverse cell types. deMULTIplex2 is further robust to low sequencing depth, suggesting the two-source contamination model broadly captures the tag distribution in multiplexing experiments.

deMULTIplex2 is built on modern statistical techniques with the EM algorithm and generalized linear model at its core. Although the EM algorithm is well known to be susceptible to local optima, we found it is surprisingly robust in deMULTIplex2, even when using a subset of down-sampled cells to train the model. The robustness in performance is achieved through deMULTIplex2’s unique modeling of tag count distributions in two separate spaces. In the first space, we model the observed contaminating tag count of negative cells as a function of total tag count, which is approximately linear in log scale. For positive cells, the tag count converges to the *y* = *x* line with increased signal-to-noise ratio, but is non-linear when ambient contamination is high. Therefore, the positive cells and the negative cells follow distinct distributions. Similarly, in the second space where we model the total contaminating tags of positive cells as a function of total tag count, the negative cells converge to the *y* = *x* line with increased total tag count, which significantly deviates from the positive cells. By fitting two GLM-NB models in these separate spaces, deMULTIplex2 is able to discriminate these distinct distributions of positive and negative cells, leading to much higher accuracy and robustness compared to those methods that rely on features of a single distribution (e.g., bimodality). We anticipate that this general approach may find utility in other types of binary classification problems. The generic model for cross-contamination employed by deMULTIplex2 may also be adapted for other labeling and multiplexing procedures, such as in lineage tracing [8, 34] and SNP-based multiplexing.

When diagnosing the model fit with Randomized Quantile Residuals, we found the RQRs approximately follow a normal distribution, suggesting the fitted model was able to explain the variance in the observed tag count. In addition, by identifying the negative cells through EM and fitting the GLM, variation in cell size was properly regressed out in the RQRs, resulting in a proper normalization for the tag count data. We anticipate this approach may be adapted for better scRNA-Seq data normalization or spatial gene expression data normalization. Finally, we provide a cosine-similarity-based initialization for the EM algorithm, which by itself better captures the bimodal distribution of tags compared to raw tag UMI counts and further improves the robustness of the algorithm.

In the deMULTIplex2 algorithm, we adopted a simple generalized linear model to approximate the relationship between the log of expected contaminating tag counts and ${\text{ln}}\left({N}_{total}+C\right)$. Although this approximation works well for many real datasets we tested, the model may work sub-optimally on even more highly heterogenous datasets with a wide range of cell size. Therefore, more sophisticated modeling, such as a better estimation of $C$—perhaps by utilizing tag counts from empty droplets—may generate even better classification performance. Another limitation of deMULTIplex2 is doublet calling, as its current doublet detection capability is not significantly better than existing methods. This is likely because deMULTIplex2 does not explicitly model the distribution of doublets. As a result, the variation in cell size and staining level can cause deMULTIplex2 to completely miss the doublets with low staining for both tags, or sometimes misclassify the doublets with low staining for one tag, and high staining for another tag as singlets. On the other hand, our benchmarking suggests no single demultiplexing method was able to robustly classify doublets. This is likely because of the limitations of tag count data, as with one single feature (just tags) per sample, it can be difficult, or even impossible to distinguish a doublet comprising one lowly-stained cell from a singlet with high tag cross-contamination. However, having multiple features (e.g., transcript counts) would bring significantly more statistical power to doublet detection, as it is highly unlikely that two sets of cell-type-specific genes, or gene programs are present within a droplet except in the doublet setting. Therefore, we recommend users to perform additional doublet detection based on gene expression data by utilizing tools such as DoubletFinder [35] and Scrublet [36].

Through the benchmarking analysis, we found that a critical factor affecting the quality of demultiplexing is the tag cross-contamination level—decreased barcode counts have a significantly smaller impact on demultiplexing efficiency. This might suggest that increased washing could be a simple and powerful way to improve demultiplexing quality. In the case of MULTI-seq, supplementing PBS washes with 1–2% BSA may function in a similar capacity to increase washing stringency. In general, deMULTIplex2’s mechanism-guided design enables the diagnosis of the sources of contamination in datasets, whether from ambient barcodes or cell-bound cross-contamination. It therefore informs improved experimental designs, such as minimizing incubation time after samples pooling before single-cell sequencing to reduce cell-bound contamination.

## Conclusions

In summary, deMULTIplex2 models the physical process of sample tag cross-contamination to correctly assign sample-of-origin in real-world sample multiplexing experiments. By applying generalized linear models and expectation–maximization, deMULTIplex2 can achieve significant performance improvement on both simulated and real-world datasets compared to existing algorithms. deMULTIplex2 helps users salvage genuine singlet cells from the non-idealized conditions encountered in real-world experiments without sacrificing classification accuracy, thus greatly improving the quality of downstream analysis.

## Methods

### Derivation of the contamination model

We model the contamination of tag counts as two components: the contamination of cell-bound tags, which is correlated with cell surface area, and the contamination from ambient floating tags captured by the droplet, which correlated with droplet size and assumed to be constant across all cells. For a particular tag B, cells can be divided into two partitions—those “positively” labeled with tag B before pooling, and those that should be “negative” for B but got contaminated by B after pooling. For each negative cell, if we denote total observed tag count as ${N}_{total}$, the ambient tag counts for each tag *k* as ${M}_{k}$, and assume cell-bound tag count of B takes a constant fraction ${p}_{B}$ of total cell-bound tag ${N}_{bound}={N}_{total}- \sum_{k=1}^{n}{M}_{k}$, then the expected contamination level of B (${\mu }_{B}$) on a negative cell can be written as

17 $$\begin{array}{c}{\mu }_{B}={p}_{B}{N}_{bound}+ {M}_{B}\\ = {p}_{B}\left({N}_{total}- \sum\limits_{k=1}^{n}{M}_{k}\right)+ {M}_{B}\\ ={p}_{B}{(N}_{total}+C),\\ where \,C=\frac{{M}_{B}}{{p}_{B}} -\sum\limits_{k=1}^{n}{M}_{k}.\end{array}$$

Taking the log transform of ${\mu }_{B}$, we can obtain the following equation:

18 $${\text{ln}}\left({\mu }_{B}\right)={\text{ln}}\left({N}_{total}+C\right)+{\text{ln}}\left({p}_{B}\right).$$

Following the argument made in the “ Results” section, we approximate the above equation with

19 $${\text{ln}}\left({\mu }_{B}\right)={\beta }_{1}^{neg}{\text{ln}}\left({N}_{total}\right)+{\beta }_{0}^{neg},$$

Assuming the observed count follows a negative binomial distribution:

20 $${X}_{B} \sim NB\left({\mu }_{B},{\theta }^{neg} \right),$$

then a negative binomial generalized linear model can be applied to the tag count of negative cells to estimate the parameters ${\beta }_{1}^{neg},{\beta }_{0}^{neg}, {\theta }^{neg}$.

For positive cells originally tagged with B, tag count B will be equal to the total tag count under ideal conditions, but often deviates from the *y* = *x* line due to contamination (Fig. 1, S1). As discussed previously, we choose to model the “contamination part” of the positive cells because the contamination of all cells happened after pooling and can be modeled uniformly. Equation 17 shows for a single contaminating tag B, its expected count follows:

$${\mu }_{B}={p}_{B}{(N}_{total}+C),$$

Then for a pool of contaminating tags,

21 $$\sum_{k\ne B}{\mu }_{k}=\sum_{k\ne B}{p}_{k}{(N}_{total}+{C}_{k}),$$

22 $$\begin{array}{c}{\text{ln}}\left(\sum\limits_{k\ne B}{\mu }_{k}\right)={\text{ln}}\left(\sum\limits_{k\ne B}{p}_{k}{(N}_{total}+{C}_{k})\right)\\ ={\text{ln}}\left(\left(1- {p}_{B}\right){N}_{total} +\sum\limits_{k\ne B}{p}_{k}{C}_{k}\right)\\ ={\text{ln}}\left({N}_{total} + \frac{\sum\limits_{k\ne B}{p}_{k}{C}_{k}}{1-{p}_{B}}\right)+{\text{ln}}\left(1-{p}_{B}\right).\end{array}$$

This result is in a form similar to Eq. 18; therefore, we can use the approximation below when fitting a GLM-NB model:

23 $${\text{ln}}(\sum_{k\ne B}{\mu }_{k}) \approx {\beta }_{1}^{pos} {\text{ln}}({N}_{total})+{\beta }_{0}^{pos},$$

24 $$\sum_{k\ne B}{X}_{k}=N_{total}-{X}_{B}\sim NB\left(\sum_{k\ne B}{\mu }_{k}, {\theta }^{pos}\right)$$

### Implementation of expectation–maximization (EM)

We implemented EM using the R programming language. We first initialize the algorithm with a non-random guess based on the cosine similarity with the canonical vector of each tag. The method then iterates between the E step and the M step to maximize the joint log likelihood until convergence or when the maximal number of iterations has been reached.

In the M step, we fit the GLM-NB on the negative cells in the $X$ vs ${N}_{total}$ space, and the positive cells in the ${N}_{total}-X$ vs ${N}_{total}$ space with the log link function, i.e.,

For negative cells:

25 $${\text{ln}}({\mu }^{neg})={\beta }_{1}^{neg}{\text{ln}}\left({N}_{total}\right)+{\beta }_{0}^{neg}$$

26 $$p\left({X}_{i}|{Z}_{i}=0\right)= \frac{\Gamma \left({X}_{i}+ {\theta }^{neg}\right)}{{X}_{i}!\Gamma \left({\theta }^{neg}\right)}{\left(\frac{{\theta }^{neg}}{{\theta }^{neg}+{\mu }^{neg} }\right)}^{{\theta }^{neg}}{\left(\frac{{\mu }^{neg}}{{\theta }^{neg}+{\mu }^{neg} }\right)}^{{X}_{i}}$$

For positive cells:

27 $${\text{ln}}({\mu }^{pos})={\beta }_{1}^{pos}{\text{ln}}\left({N}_{total}\right)+{\beta }_{0}^{pos}$$

28 $$p\left({N}_{total}-{X}_{j}|{Z}_{j}=1\right)= \frac{\Gamma \left({N}_{total}-{X}_{j}+ {\theta }^{pos}\right)}{({N}_{total}-{X}_{j})!\Gamma \left({\theta }^{pos}\right)}{\left(\frac{{\theta }^{pos}}{{\theta }^{pos}+{\mu }^{pos} }\right)}^{{\theta }^{pos}}{\left(\frac{{\mu }^{pos}}{{\theta }^{pos}+{\mu }^{pos} }\right)}^{{N}_{total}-{X}_{j}}$$

The model parameters, ${\beta }_{1}^{neg}$*, *${\beta }_{0}^{neg}$*,*${\theta }^{neg}$ and ${\beta }_{1}^{pos}$*, *${\beta }_{0}^{pos}$*,*${\theta }^{pos}$ are estimated with the *glm.nb* function from the *MASS* package.

In the E step, the posterior probabilities of the cells are calculated based on the GLM-NB predicted probability and prior probability, i.e.,

29 $$p\left({X}_{i},{Z}_{i}=1|\Theta \right)=\frac{p\left({N}_{total}-{X}_{i}|{Z}_{i}=1\right)\pi \left({Z}_{i}=1\right)}{p\left({X}_{i}|{Z}_{i}=0\right)\pi \left({Z}_{i}=0\right)+p\left({N}_{total}-{X}_{i}|{Z}_{i}=1\right)\pi \left({Z}_{i}=1\right)}$$

30 $$p\left({X}_{i},{Z}_{i}=0|\Theta \right)= 1-p\left({X}_{i},{Z}_{i}=1|\Theta \right)$$

The prior probability, $\pi \left({Z}_{i}=0\right)$ and $\pi \left({Z}_{i}=1\right)$ are defined as the fraction of the cells being negative or positive given the posterior probability from previous iteration with a 0.5 cutoff.

In the $X$ vs ${N}_{total}$ space, $p\left({X}_{i}|{Z}_{i}=0\right)$ estimated from the GLM-NB is highest around the fitted mean and becomes lower when the observed tag count deviates from the mean. However, cells with tag count lower than the fitted mean are more likely to be negative cells. Therefore, when calculating the posterior probability, we set $p\left({X}_{i}|{Z}_{i}=0\right)$ of cells below the mean to be the same as those estimated at the rounded mean in the first iteration, and to 1 in subsequent iterations. This adjustment allows more robust classification performance. In addition, we found that deMULTIplex2 does not require many cells to robustly fit its model (Fig. 2E). Thus, by default, we down-sample the positive and negative cells to a user-specified number during GLM fitting to expedite the fitting process for very large datasets. We found that when using a reasonable number of down-sampled cells, the algorithm quickly and robustly converges (Additional file 1: Fig. S2).

### Generating simulated datasets

Simulation was performed using the *simulateTags* function we built into the deMULTIplex2 package. To simulate realistic tag count data, we took a three-step approach to reproduce the labeling, pooling, and contamination process in silico. In the first step, we sample from a normal distribution to obtain the mean tag count (initial staining level) for each sample in the log scale, followed by a second sampling from a log normal distribution with previous sampled mean values and user-specified standard deviation to generate the initial tag count for all cells in each sample. This procedure resulted in a clean tag count matrix with only positive cells having non-zero entries for the corresponding tag (denoted as ${X}_{True}$). In the second step, we assume that the initial tag count is correlated with cell surface area, and use this vector as the total tag count ${N}_{total}$ in Eq. 2 to generate the expected cell-bound contamination count ${\mu }_{B}$ with user-specified ${p}_{B}$ for each tag. We then sample counts from a negative binomial distribution with the cell-specific expected contamination level ${\mu }_{B}$ and user-defined overdispersion parameter ${\theta }_{B}$ to generate the cell-bound contamination matrix (denoted as ${C}_{cell-bound}$). Finally, to simulate the ambient contamination, we perform negative binomial sampling to generate realistic ambient noise for each tag with a user-defined mean ambient contamination level, and obtain an ambient contamination matrix ${C}_{ambient}$. The singlet count matrix is then generated by summing up the three matrices ${X}_{True}$, ${C}_{cell-bound}$, and ${C}_{ambient}$. To generate doublets, we randomly sample pairs of singlets up to a user-defined percentage, and sum up the corresponding entries in ${X}_{True}$ and ${C}_{cell-bound}$. Because doublets are generally encapsulated within a single droplet, we only sample ${C}_{ambient}$ once and add the value to the simulated doublets.

For drop-out events commonly observed in scRNA-seq data, we mainly relied on the negative binomial (NB) distribution to generate the excess of 0 s, as studies have shown that NB alone is sufficient to model the amount of 0 s observed in the real-world data [37, 38]. In addition, we have built in an extra zero-inflation component to introduce even more zeros to generate more challenging scenario. For this, we follow the procedure described in ZIFA [39] and SCRABBLE [40]. Briefly, given the umi matrix simulated in the previous step, we introduce additional zeros by sampling from a Bernoulli distribution with drop-out probability ${p}_{0}={\text{exp}}(-\lambda {x}_{ij}^{2})$, where ${x}_{ij}$ is the umi count for cell *i* and tag *j*, and $\lambda$ is a user-specified exponential decay parameter. The umi count value is set to 0 (dropped out) if the sampled value is 1. In addition, we performed read down-sampling on both simulated data and real-world data to further test deMULTIplex2 and other methods’ robustness to the drop-outs under conditions of low sequencing depth, as shown in Figs. 2D and 3H,I.

For the simulated data displayed in Additional file 2: Table S1 and Fig. 2, we introduce additional variation in cell number per sample by sampling from a log normal distribution and setting a lower and upper bound on the sampled value (i.e., to prevent unrealistic extreme cell number). The average initial staining level for tags ranges from 7 to 5 in the log scale, and the overdispersion parameter of the NB distribution $\theta$ ranges from 2 to 10, which is in the range of estimated $\theta$ from real datasets (Additional file 1: Fig. S1A). For the exponential decay parameter $\lambda$ for the extra zero-inflation, we set it to 2 for Simulations 1–4, and 0.5 for Simulations 5, which corresponds to ~ 14 and ~ 61% chance of dropping a umi value of 1 to 0, respectively. For Simulation 5, since we also introduced a high level of ambient contamination and cell surface contamination, majority of the values in the umi count matrix are greater than 1. Therefore, the actual drop-out rate of this simulated dataset is lower than other simulated datasets.

Read down-sampling for Figs. 2D and 3H,I was performed using a function adapted from the *countsSampling* function in the scRecover package [41]. Briefly, for each cell, a read vector with values < 1, 2, …, *n* > (*n* = total tag count) was generated, and down-sampled uniformly to the specified number of reads. Then the down-sampled reads were partitioned to each tag based on the original tag count composition of the cell.

### Benchmarking on real datasets

To prepare public datasets for benchmarking, we preprocessed the tag count matrix from each of the studies into a uniform format and include their ground-truth labeling when available. For multi-donor datasets, we ran SNP-based sample classification using vireo [11] or souporcell [12] when such genotype-based classification were not provided.

All the methods we benchmarked were run using their default parameter setting without any parameter tuning. demuxEM and demuxmix are two methods that require information from the transcriptome. Thus, we were not able to benchmark these methods using the simulated datasets or with datasets which did not provide such information. deMULTIplex2 was also run with default parameters across all benchmarking cases, with initial cosine cutoff set to 0.5, max number of cells for GLM-NB fitting set to 5000, and max number of EM iterations set to 30.

### Supplementary Information

**Additional file 1: Fig S1.** deMULTIplex2 estimated parameters and examples of tag count distribution. **Fig S2.** Robustness of deMULTIplex2 against parameter values and down sampling. **Fig S3.** Doublet calling of deMULTIplex2 and other methods on real datasets.

**Additional file 2: Table S1.** Simulated datasets with varying size, complexity, and noise. **Table S2.** Real-world datasets used for benchmarking. **Table S3.** Performance of deMULTIplex2 and other methods on real datasets.

**Additional file 3.** Review history.

## Data Availability

Demultiplex2 is available as an R package at https://github.com/Gartner-Lab/deMULTIplex2 [42] and Zenodo (https://zenodo.org/record/8429613) [43] under the Creative Commons Attribution 4.0 International License (https://creativecommons.org/licenses/by/4.0/). The code for benchmarking deMULTIplex2 has also been deposited to github (https://github.com/Gartner-Lab/deMULTIplex2-benchmark) [44] and Zenodo (https://zenodo.org/record/8429628) [45]. Datasets used for benchmarking are all publicly available. The 8-donor PBMC MULTI-seq and SCMK dataset from McGinnis et al., 2021 [28] can be downloaded from NCBI GEO with accession number GSE161329. The 4-cell line and 8-donor PBMC datasets from Stoeckius et al., 2018 [3] can be accessed through GSE108313. The tag count matrix for 8-donor single-nucleus human brain cortex from Gaublomme et al. [4] can be accessed through docker image at https://hub.docker.com/r/regevlab/demuxem. The three batches of multi-donor bronchoalveolar lavage (BAL) datasets from Howitt et al. and Maksimovic et al. [29, 30] and the human lung cell line dataset from Howitt et al. [30] can be downloaded from https://github.com/Oshlack/hashtag-demux-paper/tree/main/data/. The PDX dataset from Winkler et al. [33] can be downloaded from NCBI GEO with accession number GSE210283.
