# Supplementary material for: deMULTIplex2: robust sample demultiplexing for scRNA-seq
Source: Genome Biol. 2024 Jan 30;25:37. doi: 10.1186/s13059-024-03177-y (PMC10829271; doi:10.1186/s13059-024-03177-y)

Supplementary Figures

Fig S1. deMULTIplex2 estimated parameters and examples of tag count distribution. (A) deMULTIplex2 estimated parameters of the GLM-NB model of negative cells for tags across 9 real datasets. (B-E) For each simulated or real dataset, a random tag is picked, and its count distributions are plotted in the two modeling spaces, colored by the deMULTIplex2-inferred posterior probability of the cells being positively tagged. The y=x line is shown in grey, and the GLM fits are shown in steel blue. RQRs are computed for both GLM-NB fits and plotted against the normal quantiles. For some cells, its RQR is infinity. These values were capped to the maximum value of non-infinity RQRs plus 1 for visualization purpose.

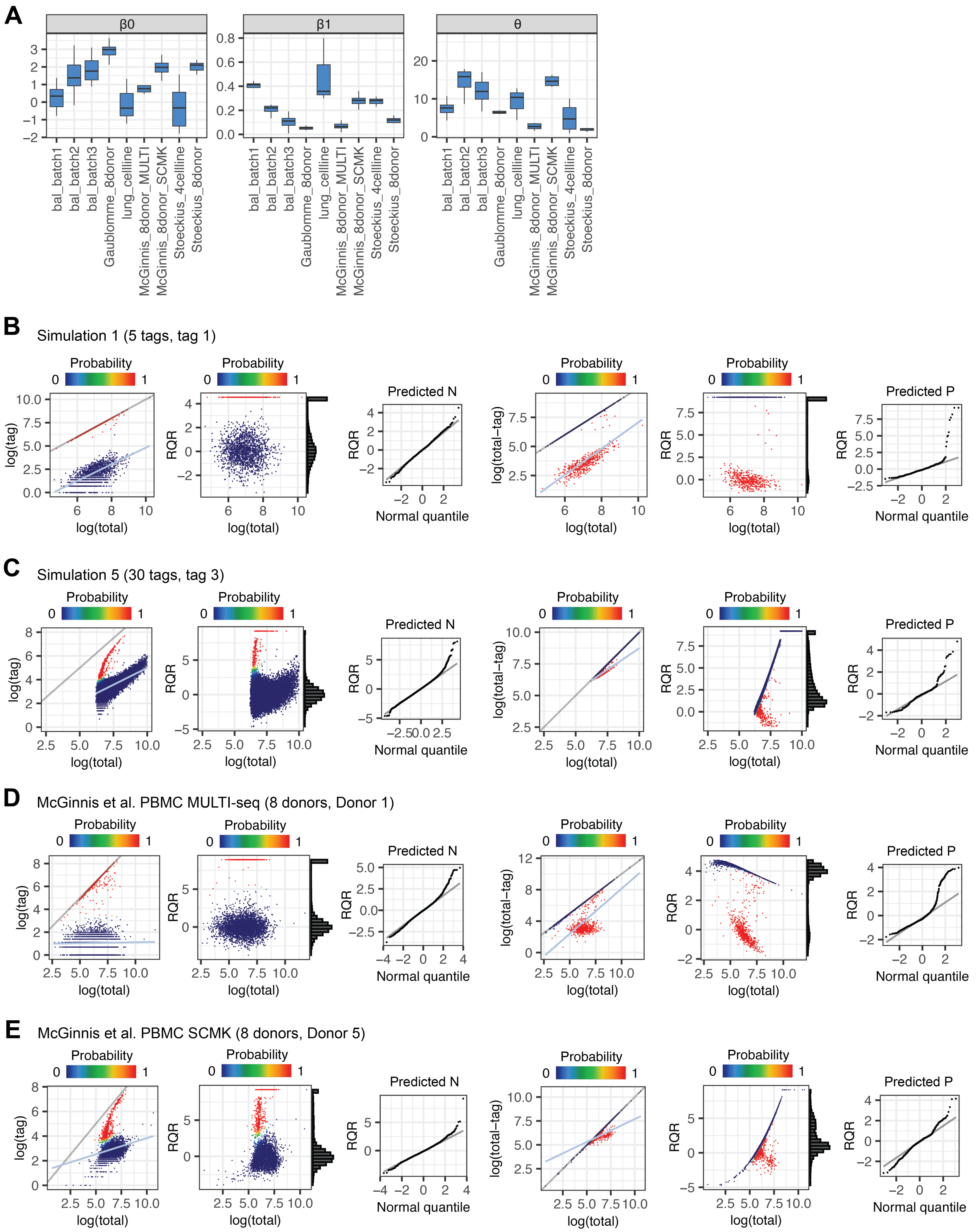
A
β0
β1
θ
bal_batch1
bal_batch2
bal_batch3
Gaublomme_8donor
lung_cellline
McGinnis_8donor_MULTI
McGinnis_8donor_SCMK
Stoeckius_4cellline
Stoeckius_8donor
B
Simulation 1 (5 tags, tag 1)
C
Simulation 5 (30 tags, tag 3)
D
McGinnis et al. PBMC MULTI-seq (8 donors, Donor 1)
E
McGinnis et al. PBMC SCMK (8 donors, Donor 5)
Probability
Predicted N
Predicted P
log(tag)
log(total−tag)
log(total)
RQR
Normal quantile

Fig S2. Robustness of deMULTIplex2 against parameter values and down-sampling. (A-C) Performance of deMULTIplex2 (measured by average F-score) on the five simulated datasets presented in Fig. 2, and the MULTI-seq and SCMK datasets from McGinnis et al. presented in Fig. 3. The line graph shows the performance of deMULTIplex2 when the EM algorithm is initialized with positive and negative cells determined by various cutoffs on cosine similarity. The heatmap shows the performance of deMULTIplex2 when the GLM-NBs were fit with down-sampled positive and negative cells and the indicated number of iterations of the EM algorithm.

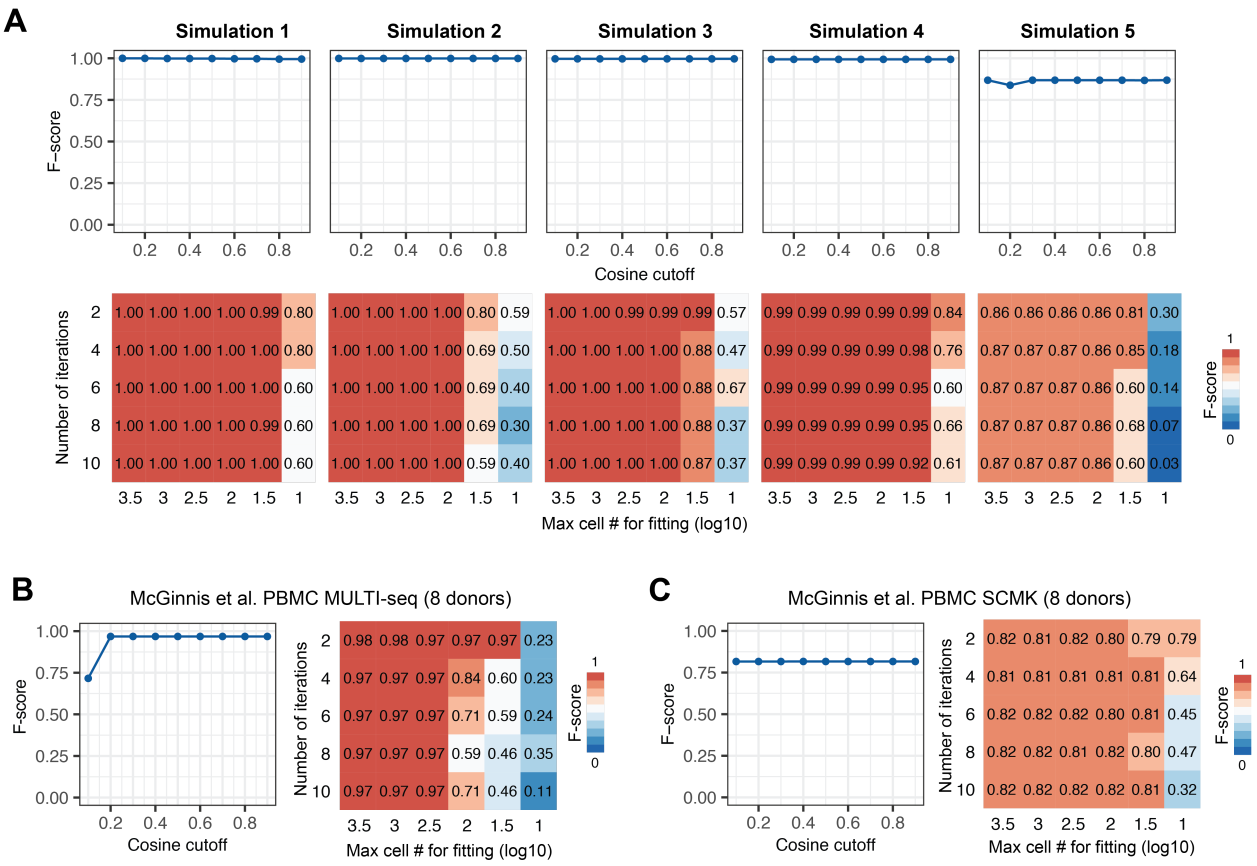

Fig S3. Doublet calling of deMULTIplex2 and other methods on real datasets. (A) Heatmap showing the fraction of true doublets called correctly by deMULTIplex2 and other methods for simulated datasets. Methods that require mRNA count matrix as input were excluded from this comparison. GMM-Demux was excluded from this analysis because the method only generates singlet classifications. (B) Heatmap showing the fraction of doublets misclassified as singlets by these methods. (C) Same as A, but shows the performance on real-world datasets. NA indicates the methods cannot be run on the corresponding datasets due to the unavailability of an mRNA count matrix or error. (D) Same as B, but shows the mis-classification on real-world datasets.

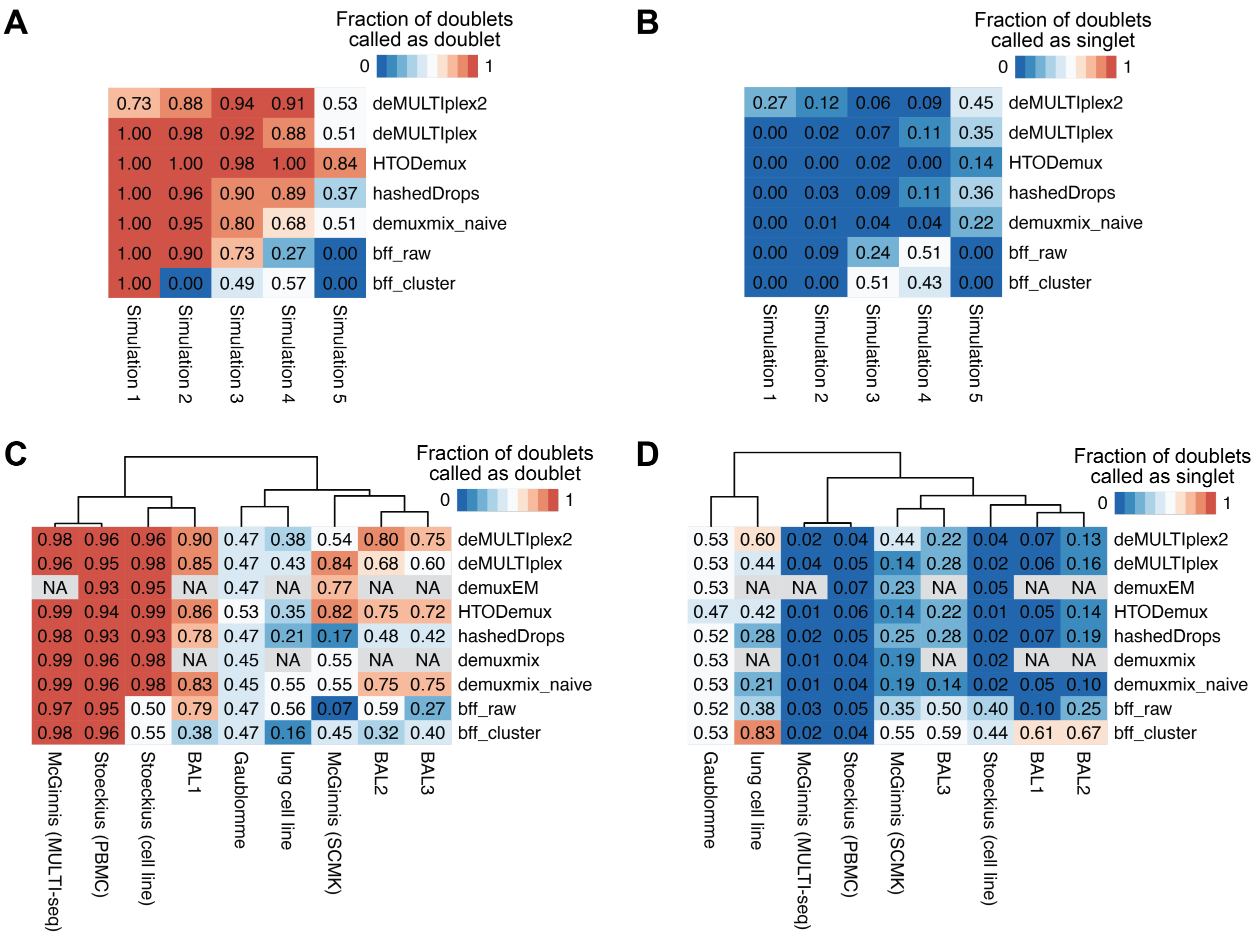

Supplement: Supplementary file 1 — Additional file 1: Fig S1. deMULTIplex2 estimated parameters and examples of tag count distribution. Fig S2. Robustness of deMULTIplex2 against parameter values and down sampling. Fig S3. Doublet calling of deMULTIplex2 and other methods on real datasets. [file 13059_2024_3177_MOESM1_ESM.pdf]
