# Supplementary material for: deMULTIplex2: robust sample demultiplexing for scRNA-seq
Source: Genome Biol. 2024 Jan 30;25:37. doi: 10.1186/s13059-024-03177-y (PMC10829271; doi:10.1186/s13059-024-03177-y)
Supplement: Supplementary file 2 — Additional file 2: Table S1. Simulated datasets with varying size, complexity, and noise. Table S2. Real-world datasets used for benchmarking. Table S3. Performance of deMULTIplex2 and other methods on real datasets. [file 13059_2024_3177_MOESM2_ESM.pdf]

Supplementary Tables

Table S1. Simulated datasets with varying size, complexity, and noise. Each dataset was generated with *simulateTags* function implemented in deMULTIplex2 with varying degree of complexity, including initial tag staining level, cell size, sample number, cell number per sample, data dispersion, cell surface contamination, ambient contamination, doublet rate and excess zero-inflation.

	Initial staining level	*Cell size variation*	*Sample number*	*Cell number per sample*	*Data dispersion (NB)*	*Cell surface contamination degree*	*Ambient contamination degree*	*Doublet rate*	*Extra zero inflation*
Simulation 1	High	Small	Low (n=5)	Balanced	Low	Low	Low	Low (2%)	Low
Simulation 2	Medium	Medium	Medium (n=10)	Medium variation	Medium	Low	Low	Low (2%)	Low
Simulation 3	Medium	Medium	Medium (n=10)	Medium variation	Medium	Medium	Medium	Medium (10%)	Low
Simulation 4	Medium	Medium	Large (n=30)	Large variation	Medium	Medium	Medium	Medium (10%)	Low
Simulation 5	Vary, low-medium	Large	Large (n=30)	Large variation	High	Medium	High	High (20%)	Medium

Table S2. Real-world datasets used for benchmarking. For each dataset, the major cell type or tissue type, relevant publication and source of data are listed. For Gaublomme et al. dataset, we obtained the tag count matrix from the docker image of demuxem.

Dataset	Tissue/Cell type	Publication	Data source
**Stoeckius (cell line)**	HEK-A, K562-B, KG1-A, THP1-C	Stoeckius et al., 2018 [1]	GSE108313
**Stoeckius (PBMC)**	Human PBMC	Stoeckius et al., 2018 [1]	GSE108313
**McGinnis (MULTI-seq)**	Human PBMC	McGinnis et al., 2021 [2]	GSE161329
**McGinnis (SCMK)**	Human PBMC	McGinnis et al., 2021 [2]	GSE161329
**Gaublomme**	Human brain cortex	Gaublomme et al., 2019 [3]	https://hub.docker.com/r/regevlab/demuxem
**Lung cancer cell line**	H1792, H3122 and H358	Howitt et al., 2022 [4]	https://github.com/Oshlack/hashtag-demux-paper/tree/main/data/cell_line_data
**BAL datasets (batch 1-3)**	Bronchoalveolar lavage fluid	Howitt et al., 2022 [4], Maksimovic et al., 2022 [4, 5]	https://github.com/Oshlack/hashtag-demux-paper/tree/main/data/BAL_data
**Winkler**	Xenograft models (PDX) of human breast cancer	Winkler et al. [6]	GSE210283

Table S3. Performance of deMULTIplex2 and other methods on real datasets. For each method, average precision, recall, and F-score of all samples are reported for each dataset. Empty values indicate the method cannot be run on the corresponding dataset due to unavailability of mRNA count matrix or an error. The top average F-score is highlighted in bold.

**McGinnis (SCMK)**	Average precision	Average recall	Average F-score
deMULTIplex2	0.969	0.739	**0.823**
deMULTIplex	0.98	0.614	0.733
demuxEM	0.979	0.627	0.729
GMM_Demux	0.125	0	0
HTODemux	0.979	0.63	0.745
hashedDrops	0.974	0.296	0.434
demuxmix	0.974	0.553	0.686
demuxmix_naive	0.974	0.553	0.686
bff_raw	0.781	0.256	0.379
bff_cluster	0.781	0.759	0.768
**McGinnis (MULTI-seq)**	Average precision	Average recall	Average F-score
deMULTIplex2	0.998	0.944	0.97
deMULTIplex	0.997	0.944	0.97
demuxEM			
GMM_Demux	0.998	0.949	0.973
HTODemux	0.998	0.879	0.935
hashedDrops	0.998	0.914	0.954
demuxmix	0.999	0.943	0.97
demuxmix_naive	0.999	0.943	0.97
bff_raw	0.998	0.957	**0.977**
bff_cluster	0.998	0.934	0.965
**Stoeckius (cell line)**	Average precision	Average recall	Average F-score
deMULTIplex2	0.993	0.934	**0.962**
deMULTIplex	0.997	0.896	0.943
demuxEM	0.992	0.603	0.746
GMM_Demux	0.981	0.87	0.922
HTODemux	0.993	0.807	0.89

hashedDrops	0.998	0.827	0.901
demuxmix	0.995	0.893	0.941
demuxmix_naive	0.996	0.892	0.941
bff_raw	0.979	0.661	0.759
bff_cluster	0.893	0.724	0.771
**Stoeckius (PBMC)**	Average precision	Average recall	Average F-score
deMULTIplex2	0.987	0.93	**0.957**
deMULTIplex	0.985	0.922	0.952
demuxEM	0.982	0.893	0.935
GMM_Demux	0.986	0.904	0.943
HTODemux	0.984	0.905	0.942
hashedDrops	0.985	0.921	0.952
demuxmix	0.988	0.922	0.954
demuxmix_naive	0.988	0.921	0.953
bff_raw	0.985	0.922	0.953
bff_cluster	0.988	0.907	0.946
**Gaublomme**	Average precision	Average recall	Average F-score
deMULTIplex2	0.97	0.982	**0.976**
deMULTIplex	0.973	0.967	0.97
demuxEM	0.973	0.97	0.971
GMM_Demux	0.974	0.958	0.965
HTODemux	0.972	0.912	0.941
hashedDrops	0.974	0.965	0.97
demuxmix	0.972	0.971	0.971
demuxmix_naive	0.972	0.967	0.969
bff_raw	0.974	0.961	0.967
bff_cluster	0.971	0.972	0.971
**Lung cancer cell line**	Average precision	Average recall	Average F-score
deMULTIplex2	0.871	0.891	**0.88**
deMULTIplex	0.854	0.497	0.621
demuxEM			
GMM_Demux	0.626	0.106	0.181
HTODemux	0.902	0.638	0.738
hashedDrops	0.885	0.568	0.663
demuxmix			
demuxmix_naive	0.945	0.62	0.743

bff_raw	0.926	0.709	0.791
bff_cluster	0.855	0.901	0.874
**BAL1**	Average precision	Average recall	Average F-score
deMULTIplex2	0.966	0.857	0.907
deMULTIplex	0.973	0.845	0.904
demuxEM			
GMM_Demux	0.977	0.796	0.876
HTODemux	0.972	0.827	0.893
hashedDrops	0.972	0.843	0.901
demuxmix			
demuxmix_naive	0.974	0.838	0.901
bff_raw	0.969	0.801	0.875
bff_cluster	0.906	0.95	**0.927**
**BAL2**	Average precision	Average recall	Average F-score
deMULTIplex2	0.898	0.774	**0.829**
deMULTIplex	0.768	0.399	0.431
demuxEM			
GMM_Demux	0.896	0.401	0.53
HTODemux	0.888	0.719	0.788
hashedDrops	0.921	0.51	0.609
demuxmix			
demuxmix_naive	0.908	0.74	0.814
bff_raw	0.687	0.481	0.537
bff_cluster	0.105	0.136	0.117
**BAL3**	Average precision	Average recall	Average F-score
deMULTIplex2	0.874	0.706	**0.774**
deMULTIplex	0.787	0.431	0.509
demuxEM			
GMM_Demux	0.936	0.337	0.484
HTODemux	0.862	0.663	0.741
hashedDrops	0.877	0.47	0.569
demuxmix			
demuxmix_naive	0.898	0.674	0.766
bff_raw	0.468	0.385	0.405
bff_cluster	0.009	0.015	0.011

**Winkler**	Average precision	Average recall	Average F-score
deMULTIplex2	0.625	0.577	**0.557**
deMULTIplex	0.559	0.439	0.438
demuxEM	0.638	0.389	0.451
GMM_Demux	0.321	0.016	0.029
HTODemux	0.601	0.377	0.423
hashedDrops	0.678	0.465	0.523
demuxmix			
demuxmix_naive			
bff_raw	0.473	0.391	0.391
bff_cluster	0.147	0.111	0.103

References

1. Stoeckius M, Zheng S, Houck-Loomis B, Hao S, Yeung BZ, Mauck WM, Smibert P, Satija R. Cell Hashing with barcoded antibodies enables multiplexing and doublet detection for single cell genomics. Genome biology. 2018;19:1-12.
2. McGinnis CS, Siegel DA, Xie G, Hartoularos G, Stone M, Ye CJ, Gartner ZJ, Roan NR, Lee SA. No detectable alloreactive transcriptional responses under standard sample preparation conditions during donor-multiplexed single-cell RNA sequencing of peripheral blood mononuclear cells. BMC biology. 2021;19:1-11.
3. Gaublomme JT, Li B, McCabe C, Knecht A, Yang Y, Drokhlyansky E, Van Wittenberghe N, Waldman J, Dionne D, Nguyen L. Nuclei multiplexing with barcoded antibodies for single-nucleus genomics. Nature communications. 2019;10:2907.
4. Howitt G, Feng Y, Tobar L, Vassiliadis D, Hickey P, Dawson MA, Ranganathan S, Shanthikumar S, Neeland M, Maksimovic J, Oshlack A. Benchmarking single-cell hashtag oligo demultiplexing methods. NAR Genomics and Bioinformatics. 2023 Dec 1;5(4):lqad086.
5. Maksimovic J, Shanthikumar S, Howitt G, Hickey PF, Ho W, Anttila C, Brown DV, Senabouth A, Kaczorowski D, Amann-Zalcenstein D, Powell JE. Single-cell atlas of bronchoalveolar lavage from preschool cystic fibrosis reveals new cell phenotypes. bioRxiv. 2022 Jun 17:2022-06.
6. Winkler J, Tan W, Diadhiou CM, McGinnis CS, Abbasi A, Hasnain S, Durney S, Atamaniuc E, Superville D, Awni L, Lee JV. Dissecting the contributions of tumor heterogeneity on metastasis at single-cell resolution. bioRxiv. 2022 Aug 5:2022-08.
