# Supplementary material for: deMULTIplex2: robust sample demultiplexing for scRNA-seq
Source: Genome Biol. 2024 Jan 30;25:37. doi: 10.1186/s13059-024-03177-y (PMC10829271; doi:10.1186/s13059-024-03177-y)
Supplement: Supplementary file 3 — Additional file 3. Review history. [file 13059_2024_3177_MOESM3_ESM.docx]

**1^st^ round**

**Reviewer 1**

Zhu et al present a novel computational method called deMULTIplex2 for multiplexing scRNA-seq data. deMULTIplex2 probabilistically infers the sample identity of each cell and classifies singlets with high accuracy. Briefly deMULTIplex2 models contamination from two sources, the cell-bound contamination, and the ambient contamination. The authors use Randomized quantile residuals (RQRs) to evaluate goodness of fit and for clustering/dimension reduction. This method seems to be appropriate and powerful in this setting. In the benchmarking analysis the authors compared their method to several competing tools and demonstrated that deMULTIplex2 outperforms existing algorithms, especially when handling large and noisy single-cell datasets or those with unbalanced sample compositions. The manuscript is well-written, easy to follow, and the statistical analyses are sound.
The overall interest may be slightly limited since the method is designed for sample multiplexing technologies and cannot be applied to more common scRNA-seq methods without sample multiplexing.
The authors did a great job on the well documented Github repo! However, the authors should consider adding the code for the benchmarking analysis as described in the manuscript.
The authors should include a table summarizing real world data sets including download links.
I enjoyed the authors' approach of analytically deriving the model. The authors use a simple chemical reaction to model the contamination. Is this the only source of contamination. Are there any bioinformatic sources of potential contamination? For example, misclassification of tags due to sequencing error? If so, the authors should discuss.

**Reviewer 2**

The manuscript introduces deMULTIplex2 as an innovative algorithm addressing challenges in single-cell sample multiplexing for scRNA-seq data. By leveraging a statistical model grounded in tag read counts and cross-contamination mechanisms, deMULTIplex2 probabilistically infers sample identity, demonstrating significant improvements over existing methods. However, it needs to be thoroughly validated, carefully benchmarked, and effectively communicated to users in order to unlock its potential benefits.

Comments
1. Can the authors please provide more explanation as to why using Randomized Quantile Residuals for model fitting? If possible, please elaborate on how this approach aids in addressing the challenges.

2. The use of probabilistic modeling and expectation-maximization might introduce computational demands, especially when dealing with large datasets. I would suggest the authors explain how to choose the appropriate down-sampling size/rate when conducting the comparison on different sizes of datasets, especially large datasets.

3. Evaluation using simulation data is insufficient in the current version. The inherent noise, doublet, and dropout events in scRNA-seq data can make it difficult to confidently assign cells to the correct samples, especially when the expression levels of genes are low or sporadic. Also, imbalanced numbers of cells across different samples can introduce biases in the demultiplexing process. Please redesign the simulation data by considering the above issues, e.g., doublet/dropout rates, gene expression levels, and number of cells, among others. More discussions are also needed as to which factor impacts the performance of each method significantly. This will help in understanding why these factors are particularly important in real-world experiments.

4. As shown in Figure 3C, a more in-depth explanation of why deMULTIplex2's performance in doublet detection is comparable to existing methods could be helpful.

5. What are the logical next steps after establishing the superiority of deMULTIplex2? Please discuss more about the potential areas for improvement or expansion that would enhance the manuscript.

**Authors’ response**

Reviewer #1: Zhu et al present a novel computational method called deMULTIplex2 for multiplexing scRNA-seq data. deMULTIplex2 probabilistically infers the sample identity of each cell and classifies singlets with high accuracy. Briefly deMULTIplex2 models contamination from two sources, the cell-bound contamination, and the ambient contamination. The authors use Randomized quantile residuals (RQRs) to evaluate goodness of fit and for clustering/dimension reduction. This method seems to be appropriate and powerful in this setting. In the benchmarking analysis the authors compared their method to several competing tools and demonstrated that deMULTIplex2 outperforms existing algorithms, especially when handling large and noisy single-cell datasets or those with unbalanced sample compositions. The manuscript is well-written, easy to follow, and the statistical analyses are sound. 
The overall interest may be slightly limited since the method is designed for sample multiplexing technologies and cannot be applied to more common scRNA-seq methods without sample multiplexing. 
The authors did a great job on the well documented Github repo! However, the authors should consider adding the code for the benchmarking analysis as described in the manuscript.
The authors should include a table summarizing real world data sets including download links.
I enjoyed the authors' approach of analytically deriving the model. The authors use a simple chemical reaction to model the contamination. Is this the only source of contamination. Are there any bioinformatic sources of potential contamination? For example, misclassification of tags due to sequencing error? If so, the authors should discuss.

**We thank the reviewer for the constructive feedback. We have revised the manuscript accordingly, and have performed additional analysis to address the question on other sources of potential contamination, please see our detailed response below:**

1. “The overall interest may be slightly limited since the method is designed for sample multiplexing technologies and cannot be applied to more common scRNA-seq methods without sample multiplexing.”

**With the increasing throughput of single cell technology, sample multiplexing technologies are routinely applied to single cell sequencing workflows. Since the first introduction of this technology in 2018, sample multiplexing papers have been cited over 3500 times. This technology has also been incorporated into commercial single cell kits such as the 10x Genomics 3’ CellPlex kit and the BD® single-cell multiplexing kit. In our experience, existing demultiplexing methods do not fully leverage the power of sample multiplexing technologies due to false negatives and positives. Therefore, there is a need for accurate and scalable sample demultiplexing for the increasing number of datasets generated with this technology.**

1. “However, the authors should consider adding the code for the benchmarking analysis as described in the manuscript.”

**As requested, we have deposited all our benchmarking codes to the github repo (**[**https://github.com/Gartner-Lab/deMULTIplex2-benchmark**](https://github.com/Gartner-Lab/deMULTIplex2-benchmark)**), as well as Zenodo (**[**https://zenodo.org/record/8429628**](https://zenodo.org/record/8429628)**).**

2. “The authors should include a table summarizing real world data sets including download links.”

**We have summarized all real-world datasets in Table S2 in the manuscript, also shown below.**

**Table S2. Real-world datasets used for benchmarking.**

| **Dataset** | **Tissue/Cell type** | **Publication** | **Data source** |
| --- | --- | --- | --- |
| **Stoeckius (cell line)** | HEK-A, K562-B, KG1-A, THP1-C | Stoeckius et al., 2018 [1] | GSE108313 |
| **Stoeckius (PBMC)** | Human PBMC | Stoeckius et al., 2018 [1] | GSE108313 |
| **McGinnis (MULTI-seq)** | Human PBMC | McGinnis et al., 2021 [2] | GSE161329 |
| **McGinnis (SCMK)** | Human PBMC | McGinnis et al., 2021 [2] | GSE161329 |
| **Gaublomme** | Human brain cortex | Gaublomme et al., 2019 [3] | https://hub.docker.com/r/regevlab/demuxem |
| **Lung cancer cell line** | H1792, H3122 and H358 | Howitt et al., 2022 [4] | https://github.com/Oshlack/hashtag-demux-paper/tree/main/data/cell_line_data |
| **BAL datasets (batch 1-3)** | Bronchoalveolar lavage fluid | Howitt et al., 2022 [4], Maksimovic et al., 2022 [4, 5] | https://github.com/Oshlack/hashtag-demux-paper/tree/main/data/BAL_data |
| **Winkler** | Xenograft models (PDX) of human breast cancer | Winkler et al. [6] | GSE211145 |

4. “I enjoyed the authors' approach of analytically deriving the model. The authors use a simple chemical reaction to model the contamination. Is this the only source of contamination. Are there any bioinformatic sources of potential contamination? For example, misclassification of tags due to sequencing error? If so, the authors should discuss.”
**With current sequencing technology, the majority of reads will have Phred quality scores (Q score) > 30, meaning less than 1 in 1000 error rate [7]. Many single-cell multiplexing technologies also have distinct tags with different oligonucleotide sequences. For example, MULTI-Seq tags have a minimal hamming distance of 4 (Fig. R1A). Thus, it is relatively unlikely that sequencing error will lead to significant number of mis-mapped reads. On the other hand, current oligonucleotide synthesis technology has a higher error rate, which can cause a small proportion of tags having incorrect bases during synthesis. As a result, if two tags are not carefully designed to be distinct, or if the read mapping is not stringent, it is possible for some reads that originate from one sample tag to “leak” into the signal of another similar tag. We performed the following *in silico* experiment to analyze the effect of mis-mapped reads on the classification accuracy of deMULTIplex2 and other methods.**

**In deMULTIplex2, a user can specify the stringency of tag read mapping by including reads with x hamming distances from the reference sequence. By changing x from 0 to 3 for the MULTI-seq dataset by Mcginnis et al [2], we could simulate situation where more contaminating reads get included for each tag due to relaxation of read mapping stringency, or more broadly, due to synthesis error or sequencing error (Fig. R1B). The performance of all methods was not affected with up to 2 bases mismatches. However, when including reads with up to 3 bases mismatches, there is a significant drop in performance for all methods except deMULTIplex2. Further analysis suggests the deteriorating performance may be due to leak of signal from tags with synthesis error. For example, a small amount of B2 and B4 cells have slight “positive” signal for A10 (Fig. R1C), likely because these reads originate from B2 and B4 (with some synthesis error), but gets mapped to A10 because of the relaxation on mapping stringency. Under such circumstances, deMULTIplex2 was able to confidently detect the difference between true A10 positive cells and the negative cells, likely because the distributions of positive and negative cells are very distinct in the second space where we model the total contaminating tags of positive cells as a function of total tag count (Fig. R1D). Thus, this *in silico* experiment demonstrates deMULTIplex2’s robustness against cross-contamination of tags due to oligo synthesis error or sequencing error.**

**Figure R1.** **Benchmarking deMULTIplex2’s performance against cross-contamination of tags due to read mapping error.** (A) Hamming distance between MULTI-seq tags used in Mcginnis et al [2]. (B) Performance of deMULTIplex2 and other methods when including reads with varying number of mismatches to the reference sequences. (C) Signal of A10 when including reads with max hamming distance to the reference equal to 3. Points are colored by ground-truth labels. (D) Same plots as C, with points colored by deMULTIplex2 inferred positive probability for A10. The y=x line (grey) and the GLM-NB fit (steel blue line) are also plotted.

Reviewer #2:
“The manuscript introduces deMULTIplex2 as an innovative algorithm addressing challenges in single-cell sample multiplexing for scRNA-seq data. By leveraging a statistical model grounded in tag read counts and cross-contamination mechanisms, deMULTIplex2 probabilistically infers sample identity, demonstrating significant improvements over existing methods. However, it needs to be thoroughly validated, carefully benchmarked, and effectively communicated to users in order to unlock its potential benefits.”

**We thank the reviewer for the excellent suggestions. We have revised our manuscript accordingly, especially in re-designing our simulation model to encompass a wider range of realistic conditions. Please see our response to each of the comments below.**

Comments
1.    “Can the authors please provide more explanation as to why using Randomized Quantile Residuals for model fitting? If possible, please elaborate on how this approach aids in addressing the challenges.”

**To clarify, Randomized Quantile Residuals were not used for model fitting for deMULTIplex2, but only for model validation and UMAP visualization. Residual analysis is commonly used for diagnosing the goodness-of-fit (GOF) for generalized linear models. Typically, Pearson residuals and deviance residuals are used [8, 9]. However, for discrete random variables, such as RNA-Seq count data, these residuals are far from normal and cannot be used for model diagnostics. RQR overcomes this issue by introducing randomization to produce continuously distributed residuals. Theoretically, the RQRs converges to standard normal if the model parameters are consistently estimated. Therefore, several recent studies have adopted this strategy to diagnose regression models [10, 11], including scTransform [12, 13], a popular method for scRNA-seq data normalization. We also use RQRs for UMAP visualization because it corrects for the linear dependence on total tag count and stabilizes the variance for small counts, as pointed out by a recent review paper [14].**

2.    “The use of probabilistic modeling and expectation-maximization might introduce computational demands, especially when dealing with large datasets. I would suggest the authors explain how to choose the appropriate down-sampling size/rate when conducting the comparison on different sizes of datasets, especially large datasets.”

**The reviewer raises a valid concern which is a common case for probabilistic modeling with large datasets. In deMULTIplex2, we adopted the generalized linear model, which is an efficient algorithm with complexity equal to** O(p3+Np2) **[15], or** O(N) **with p=1 (single predictor: log of total tag count) and N being the number of cells. In addition, as the EM almost always converges within very few iterations (updated Figure S2), the overall complexity of the deMULTIplex2 algorithm scales linearly with the number of cells. In practice, we were able to run all benchmarking datasets on a macbook laptop in couple of minutes even without performing any down-sampling.**

**We allow users to perform down-sampling because deMULTIplex2 does not need a lot of cells to learn the distribution of the global dataset. We demonstrate this with two cell down-sampling experiments. In the first experiment, we directly down-sample five simulated datasets to retain different number of cells (Figure 2E) and benchmarked deMULTIplex2’s performance on the *down-sampled* datasets. The overall performance, as quantified by F-score, shows deMULTIplex2 has maintained its classification accuracy even with 1-5% of original cells (or ~10 cells per group), while several other methods exhibited significant deteriorating performance with decreased number of cells. This suggest deMULTIplex2’s model fitting does not heavily rely on the cell number. In the second experiment, we performed cell down-sampling when fitting the GLM, and benchmarked deMULTIplex2’s performance on *all cells* in the *original* dataset. As shown in Figure S2, deMULTIplex2 is capable of accurately classifying *all cells* even when the model fitting was performed with about 100 to 300 cells sampled from the positive and negative population. This suggest that the model deMULTIplex2 constructed from relatively few cells can be used to correctly predict the identity of all cells in the dataset.**

**We next asked how the uncertainty due to random sampling translates to the uncertainty in the final classification result. On the MULTI-seq dataset by Mcginnis et al [2], we repeated random down-sampling of cells 100 times at each down-sampling rate and measured the distribution of the estimated GLM coefficients for a randomly selected barcode (Fig R2A). The estimation of the GLM coefficients converges to the expected value with ~300 cells. However, there are still some variations in the estimated GLM coefficients, but such variation does not seem to affect the overall performance of deMULTIplex2 (Fig. S2B). This suggests the posterior probability of each cell has much smaller variation, likely because it’s calculated by integrating the probabilities calculated from separate fit of GLM on the positive and negative cells. Indeed, almost no variance was observed in posterior probability for most of the cells (Fig. R2B), suggesting they were consistently and robustly classified. There exist a few cells (likely near the decision boundary) with high posterior probability variance when the number of sampled cells are low. Increasing sampling rate significantly improves the classification of these cells.**

**Figure 2.** (E) Performance of all methods on the simulated datasets with down-sampled cells.

**Figure S2. Robustness of deMULTIplex2 against parameter values and down-sampling.** (A-C) Performance of deMULTIplex2 (measured by average F score) on the five simulated datasets presented in Fig. 2, and the MULTI-seq and SCMK datasets from McGinnis et al. presented in Fig. 3 [28]. The line graph shows the performance of deMULTIplex2 when the EM algorithm is initialized with positive and negative cells determined by various cutoffs on cosine similarity. The heatmap shows the performance of deMULTIplex2 when the GLM-NBs were fit with down-sampled positive and negative cells and the indicated number of iterations of the EM algorithm.

**Figure R2. Variation in GLM model fitting and posterior probability calculation.** (A) Distribution of estimated coefficients for true negative cells and true positive cells of Barcode 12 for 100 random down-samplings. (B) Standard deviation of the posterior probability for each cell for 100 random down-samplings. Hierarchical clustering was performed for the cells with the Ward's clustering criterion.

3.    “Evaluation using simulation data is insufficient in the current version. The inherent noise, doublet, and dropout events in scRNA-seq data can make it difficult to confidently assign cells to the correct samples, especially when the expression levels of genes are low or sporadic. Also, imbalanced numbers of cells across different samples can introduce biases in the demultiplexing process. Please redesign the simulation data by considering the above issues, e.g., doublet/dropout rates, gene expression levels, and number of cells, among others. More discussions are also needed as to which factor impacts the performance of each method significantly. This will help in understanding why these factors are particularly important in real-world experiments.”

**We have redesigned the simulation function to address the reviewer’s concerns. The current simulation function is capable of simulating count distributions very close to the real-world datasets with varying degrees of complexity, including initial tag staining level, cell size, sample number, cell number per sample, data dispersion, cell surface contamination, ambient contamination, doublet rate and excess zero-inflation (Table 1, Figure 2, Figure S1B-E and Methods).**

**To clarify, only the tag count matrix, but not the gene expression matrix, is used for sample classification by deMULTIplex2. Most single cell multiplexing technologies aim to tag the surface of the cell in order to prevent the potential confounding impact of tags on gene expression. There may be association between the number of expressed genes and cell surface size (Fig. 1B), but our analysis suggests that the total tag umi count of a cell is a better approximation of the available cell surface for tagging, thus, we use this measurement for modeling the distribution of tag counts (Equation 1 and 2). Therefore, in the current simulation, we simulate a range of total tag count to reflect intrinsic variations in cell size and tag staining level, which we hope is what the reviewer is suggesting by “gene expression levels”.**

**For drop-out events in scRNA-seq data, we mainly relied on the negative binomial (NB) distribution to generate the excess of 0s, as studies have shown that NB alone is sufficient to model the amount of 0s observed in the real-world data [13, 16]. In the current simulation, we have also built in an extra zero-inflation component to introduce even more zeros to generate more challenging scenario. For this, we follow the procedure described in ZIFA [17] and SCRABBLE [18]. In addition, we performed read down-sampling on both simulated data and real-world data to further test deMULTIplex2’s robustness to the drop-outs under conditions of low sequencing depth, as shown in Fig. 2D, Fig. 3H-I.**

**By better modeling realistic factors affecting multiplexing experiment, our new simulation function can serve as a powerful tool for systematically benchmarking existing methods. However, our preliminary test runs suggest the influence of each parameter may be non-linear, non-independent, and there can be interactions between these factors leading to an even greater impact. We feel it is beyond of the scope of the current manuscript to systematically test the impact of each factor or combination of factors for all methods. Therefore, we generated five simulation datasets with varying data size, complexity and noise covering a wide span of possible realistic parameter values (Table S1, Fig. 2A-C). We were able to learn quite a few interesting things from the new simulations.**

**Consistent with previous observation, almost all methods perform relatively well on small and clean dataset (Fig. 2B, Simulation 1). Changes in data composition (sample number, cell number per sample etc.) have limited impact on the performance of most methods when the cross-contamination level is low (Fig. 2B, Simulation 2 vs Simulation 1). Similarly, on small-to-medium-sized data, elevated level of cross-contamination and doublet rate only modestly decreases the accuracy of most methods (Fig. 2B, Simulation 3 vs Simulation 2). Notably, deMULTIplex2 was the only method that maintained its top performance across these conditions (Fig. 2B).**

**We observed different results in more complex simulations. For example, when the sample number increased from 10 to 30 and imbalance in cell number across samples  was increased, a dramatic drop in performance was observed for multiple methods (Simulation 4 vs Simulation 3, Fig. 2B). Methods such as GMM-Demux and BFF completely fail to recover many samples, likely because these methods heavily rely on the assumption of bimodal distribution, which is not a realistic assumption for large datasets (Figure 2A, bottom panel). Methods such as HTODemux and demuxmix (naïve mode) have a significant drop in recall despite maintaining their precision (Fig. 2C), mainly because these methods were classifying the high-tag-count, high-confidence cells. In theory, achieving high recall without sacrificing precision is much more challenging because higher recall requires the method to draw a decision boundary closer to the ambiguous and negative territory (without crossing the border to incur misclassification). In practice, high recall is often desired because it means many more real singlets can be recovered from expensive single cell experiments. In this case, deMULTIplex2 was able to correctly identify the close-to-optimal decision boundary (Fig. 2A, bottom panel) and classified >99% real singlets, while HTOdemux and demuxmix (naïve mode) were only able to achieve an average recall of 52% and 73%, respectively. Upon further increasing the data complexity and noise of the simulation, all methods suffer in performance, but deMULTIplex2 maintained its top performance and has consistent classification accuracy across all samples, even for those with very few cells (Simulation 5, Fig. 2A, C).**

**For each of these simulated datasets, we performed read down-sampling to benchmark the methods’ robustness to sequencing depth. As shown in Fig. 2D, deMULTIplex2 was consistently the top performer across a range of sequencing depths, with several methods failing when the total tag count per cell became too low and the drop-out rate became too high (Fig 2D). This suggests that the statistical model powering deMULTIplex2 is robust against low sequencing depth and high drop-out rate. The sequencing depth has the most significant impact on performance for large and noisy datasets, suggesting increasing sequencing depth (before saturation) may be beneficial for these types of data.**

**We have included the relevant discussions in the main text.**

**Table S1. Simulated datasets with varying size, complexity and noise.**

|  | *Initial staining level* | *Cell size variation* | *Sample number* | *Cell number per sample* | *Data dispersion (NB)* | *Cell surface contamination degree* | *Ambient contamination degree* | *Doublet rate* | *Extra zero inflation* |
| --- | --- | --- | --- | --- | --- | --- | --- | --- | --- |
| *Simulation 1* | High | Small | Low (n=5) | Balanced | Low | Low | Low | Low (2%) | Low |
| *Simulation 2* | Medium | Medium | Medium (n=10) | Medium variation | Medium | Low | Low | Low (2%) | Low |
| *Simulation 3* | Medium | Medium | Medium (n=10) | Medium variation | Medium | Medium | Medium | Medium (10%) | Low |
| *Simulation 4* | Medium | Medium | Large (n=30) | Large variation | Medium | Medium | Medium | Medium (10%) | Low |
| *Simulation 5* | Vary, low-medium | Large | Large (n=30) | Large variation | High | Medium | High | High (20%) | Medium |

**Figure 2. Performance of deMULTIplex2 on simulated datasets.** (A) Five simulations with different data size, complexity and noise, as listed in Table 1. The top row shows UMAPs computed with raw tag UMI count. The middle row shows UMAPs based on deMULTIplex2-computed RQRs. The bottom row shows the cell distribution in the log(tag umi) vs log(total tag count) space of a randomly selected tag in each simulation, colored by ground-truth identity and deMULTIplex2 computed posterior probability of being positive for that tag. The y=x line (grey) and the GLM-NB fit for the negative cells (steel blue line) are also plotted. (B) Heatmap summarizing the F score of deMULTIplex2 and other methods on the five simulated datasets. Methods that require mRNA count matrix as input were excluded from this comparison. NA indicate the method failed to run. (C) Per-tag performance of deMULTIplex2 compared to other methods. Mean values are highlighted with the diamond points. (D) Performance of all methods on the simulated datasets with down-sampled reads. (E) Performance of all methods on the simulated datasets with down-sampled cells.

4. “As shown in Figure 3C, a more in-depth explanation of why deMULTIplex2's performance in doublet detection is comparable to existing methods could be helpful.”

**With the updated simulation, we were also able to benchmark deMULTIplex2 and other methods’ doublet classification across data sets having various sizes, complexities, and noise conditions. As shown in Figure S3A-B, deMULTIplex2 had the top doublet detection performance when the doublet rate and noise level was medium (10%), but did not perform as well as several other methods when the doublet rate was very low (2%) or very high (20%). The instability in deMULTIplex2’s doublet detection capability is mainly because we did not explicitly model the doublet distribution in our model. As illustrated in Fig 1E-F, the doublets often lie between the positive singlets and the negative cells, covering a wide span of space. Under most circumstances, deMULTIplex2 was able to correctly classify those doublets close to the positive singlet as “positive”, but may miss the doublets with a lower level of staining (close to the negative cells). Therefore, the variation in cell size and staining level can cause deMULTIplex2 to completely miss the doublets with low staining for both tags, or sometimes misclassify the doublets with low staining for one tag, and high staining for another tag as singlets. On the other hand, our benchmarking suggests no single demultiplexing method was able to robustly classify doublets. This is likely because of the limitations of tag count data, as with one single feature (just tags) per sample, it can be difficult, or even impossible to distinguish a doublet comprising one lowly-stained cell from a singlet with high tag cross-contamination. However, having multiple features (e.g., transcript counts), would bring significantly more statistical power to doublet detection, as it is highly unlikely that two sets of cell type-specific genes, or gene programs are present within a droplet except in the doublet setting. Therefore, we recommend users to perform additional doublet detection based on gene expression data by utilizing tools such as DoubletFinder [19] and Scrublet [20], as doublets formed by two distinct cell types or cell states can be more readily identified due to the presence of distinct cell-type-specific genes (or gene programs). We will continue to work on deMULTIplex2 to improve its doublet classification capability in the future, but we feel further improvements on this front are beyond the scope of this work.**

**Figure S3. Doublet calling of deMULTIplex2 and other methods on real datasets.**  (A) Heatmap showing the fraction of true doublets called correctly by deMULTIplex2 and other methods for simulated datasets. Methods that require an mRNA count matrix as input were excluded from this comparison. GMM-Demux was excluded from this analysis because the method only generates singlet classifications. (B) Heatmap showing the fraction of doublets misclassified as singlets by these methods. (C) Same as A, but shows the performance on real-world datasets. NA indicates the methods failed to run on the corresponding datasets due to the unavailability of an mRNA count matrix or error. (D) Same as B, but shows the mis-classification on real-world datasets.

5.  “What are the logical next steps after establishing the superiority of deMULTIplex2? Please discuss more about the potential areas for improvement or expansion that would enhance the manuscript.”

**As discussed above, we will continue improving deMULTIplex2’s ability to identify doublets, and also develop a pipeline for seamlessly bridging deMULTIplex2 and DoubletFinder [19] for scRNA-Seq data preprocessing. In the current deMULTIplex2 algorithm, we adopted a simple generalized linear model to approximate the linear relationship between log of expected contaminating tag counts and** ln Ntotal+C **. Although the approximation works well for many real datasets we tested, the model may work sub-optimally on highly heterogenous datasets with a wide range of cell size. Therefore, more sophisticated modeling, such as a better estimation of** C **– perhaps by utilizing tag counts from empty droplets – may generate even better classification performance. We will continue to improve deMULTIplex2’s classification capability in this direction.**

**We found that by properly modeling the “positive” cells and “negative” cells separately, we can reach very high classification accuracy and robustness compared to those methods that rely on features of a single distribution (e.g., bimodality). This general approach may find utility in other types of binary classification problems. The generic model for cross-contamination employed by deMULTIplex2 may also be adapted for other labeling and multiplexing procedures, such as in lineage tracing [21, 22] and SNP-based multiplexing.**

**In addition, by identifying the negative cells through EM and fitting the GLM, variation in cell size was properly regressed out in the RQRs, resulting in a proper normalization for the tag count data. We anticipate this approach may be adapted for better scRNA-Seq data normalization or spatial gene expression data normalization.**

**We have added a discussion of these points to the main manuscript.**

1.	Stoeckius M, Zheng S, Houck-Loomis B, Hao S, Yeung BZ, Mauck WM, Smibert P, Satija R. Cell Hashing with barcoded antibodies enables multiplexing and doublet detection for single cell genomics. Genome biology. 2018;19**:**1-12.

2.	McGinnis CS, Siegel DA, Xie G, Hartoularos G, Stone M, Ye CJ, Gartner ZJ, Roan NR, Lee SA. No detectable alloreactive transcriptional responses under standard sample preparation conditions during donor-multiplexed single-cell RNA sequencing of peripheral blood mononuclear cells. BMC biology. 2021;19**:**1-11.

3.	Gaublomme JT, Li B, McCabe C, Knecht A, Yang Y, Drokhlyansky E, Van Wittenberghe N, Waldman J, Dionne D, Nguyen L. Nuclei multiplexing with barcoded antibodies for single-nucleus genomics. Nature communications. 2019;10**:**2907.

4.	Howitt G, Feng Y, Tobar L, Vassiliadis D, Hickey P, Dawson MA, Ranganathan S, Shanthikumar S, Neeland M, Maksimovic J. Benchmarking single-cell hashtag oligo demultiplexing methods. bioRxiv. 2022**:**2022.2012. 2020.521313.

5.	Maksimovic J, Shanthikumar S, Howitt G, Hickey PF, Ho W, Anttila C, Brown DV, Senabouth A, Kaczorowski D, Amann-Zalcenstein D. Single-cell atlas of bronchoalveolar lavage from preschool cystic fibrosis reveals new cell phenotypes. bioRxiv. 2022**:**2022.2006. 2017.496207.

6.	Winkler J, Tan W, Diadhiou CM, McGinnis CS, Abbasi A, Hasnain S, Durney S, Atamaniuc E, Superville D, Awni L. Dissecting the contributions of tumor heterogeneity on metastasis at single-cell resolution. bioRxiv. 2022**:**2022.2008. 2004.502697.

7.	Manley LJ, Ma D, Levine SS. Monitoring error rates in Illumina sequencing. Journal of biomolecular techniques: JBT. 2016;27**:**125.

8.	Agresti A. *Categorical data analysis.* John Wiley & Sons; 2012.

9.	McCullagh P, Nelder JA. Generalized linear models 2nd edition chapman and hall. London, UK. 1989.

10.	Bai W, Dong M, Li L, Feng C, Xu W. Randomized quantile residuals for diagnosing zero-inflated generalized linear mixed models with applications to microbiome count data. BMC bioinformatics. 2021;22**:**1-18.

11.	Feng C, Li L, Sadeghpour A. A comparison of residual diagnosis tools for diagnosing regression models for count data. BMC Medical Research Methodology. 2020;20**:**1-21.

12.	Hafemeister C, Satija R. Normalization and variance stabilization of single-cell RNA-seq data using regularized negative binomial regression. Genome biology. 2019;20**:**296.

13.	Choudhary S, Satija R. Comparison and evaluation of statistical error models for scRNA-seq. Genome biology. 2022;23**:**27.

14.	Ahlmann-Eltze C, Huber W. Comparison of transformations for single-cell RNA-seq data. Nature Methods. 2023**:**1-8.

15.	[<https://docs.h2o.ai/h2o/latest-stable/h2o-docs/data-science/glm.html>]

16.	Kim TH, Zhou X, Chen M. Demystifying “drop-outs” in single-cell UMI data. Genome biology. 2020;21**:**196.

17.	Pierson E, Yau C. ZIFA: Dimensionality reduction for zero-inflated single-cell gene expression analysis. Genome Biology. 2015;16**:**1-10.

18.	Peng T, Zhu Q, Yin P, Tan K. SCRABBLE: single-cell RNA-seq imputation constrained by bulk RNA-seq data. Genome biology. 2019;20**:**1-12.

19.	McGinnis CS, Murrow LM, Gartner ZJ. DoubletFinder: doublet detection in single-cell RNA sequencing data using artificial nearest neighbors. Cell systems. 2019;8**:**329-337. e324.

20.	Wolock SL, Lopez R, Klein AM. Scrublet: computational identification of cell doublets in single-cell transcriptomic data. Cell systems. 2019;8**:**281-291. e289.

21.	Biddy BA, Kong W, Kamimoto K, Guo C, Waye SE, Sun T, Morris SA. Single-cell mapping of lineage and identity in direct reprogramming. Nature. 2018;564**:**219-224.

22.	Guo C, Kong W, Kamimoto K, Rivera-Gonzalez GC, Yang X, Kirita Y, Morris SA. CellTag Indexing: genetic barcode-based sample multiplexing for single-cell genomics. Genome biology. 2019;20**:**1-13.

**2^nd^ round**

**Reviewer 1**

The authors have done a great job addressing my concerns.

**Reviewer 2**

The authors have addressed all my concerns. I have no further comments.
